# Target identification reveals protein arginine methyltransferase 1 is a potential target of phenyl vinyl sulfone and its derivatives

**DOI:** 10.1042/BSR20171717

**Published:** 2018-04-20

**Authors:** Cheng-Han Yu, Chi-Chi Chou, Der-Yen Lee, Kay-Hooi Khoo, Geen-Dong Chang

**Affiliations:** 1 Graduate Institute of Biochemical Sciences, College of Life Science, National Taiwan University, Taipei 10617, Taiwan; 2 Institute of Biological Chemistry, Academia Sinica, Taipei 11529, Taiwan; 3 Graduate Institute of Integrated Medicine, China Medical University, Taichung 40402, Taiwan

**Keywords:** Bay11-7082, covalent drug, phenyl vinyl sulfone, phenyl vinyl sulfonate, protein arginine methyltransferase 1, target identification

## Abstract

Phenyl vinyl sulfone (PVS) and phenyl vinyl sulfonate (PVSN) inactivate protein tyrosine phosphatases (PTPs) by mimicking the phosphotyrosine structure and providing a Michael addition acceptor for the active-site cysteine residue of PTPs, thus forming covalent adducts between PVS (or PVSN) and PTPs. We developed a specific antiserum against PVS. This antiserum can be used in general antibody-based assays such as immunoblotting, immunofluorescence staining, and immunoprecipitation. Target identification through immunoprecipitation and mass spectrometry analysis reveals potential targets of PVS, mostly proteins with reactive cysteine residues or low-p*K*_a_ cysteine residues that are prone to reversible redox modifications. Target identification of PVSN has been conducted because the anti-PVS antiserum can also recognize PVSN. Among the targets, protein arginine methyltransferase 1 (PRMT1), inosine-5′-monophosphate dehydrogenase 1, vimentin, and glutathione reductase (GR) were further confirmed by immunoprecipitation followed by immunoblotting. In addition, PVSN and Bay11-7082 inhibited GR activity, and PVS, PVSN, and Bay 11-7082 inhibited PRMT1 activity in *in vitro* assays. In addition, treatment of PVSN, Bay11-7082, or Bay 11-7085 in cultured HeLa cells can cause the quick decline in the levels of protein asymmetric dimethylarginine. These results indicate that the similar moiety among PVS, PVSN, Bay 11-7082, and Bay 11-7085 can be the key structure of lead compounds of PRMT1. Therefore, we expect to use this approach in the identification of potential targets of other covalent drugs.

## Introduction

Protein tyrosine phosphorylation, dynamically controlled by the activities of protein tyrosine kinases (PTKs) and protein tyrosine phosphatases (PTPs), is critical in the regulation of cell proliferation, differentiation, metabolism, and survival. Overall, there are over 100 human PTP-superfamily genes that can be classified into the classical phosphotyrosine-specific phosphatases and the dual specificity phosphatases [1]. The classical PTPs possess an active-site site motif HCX_5_R, in which the cysteine sulfhydryl group deprotonates easily due to its low p*K*_a_ and functions as a nucleophile for the enzymatic catalysis [2,3]. The low p*K*_a_ property of the catalytic cysteine residue also renders PTPs susceptible to oxidation and transient inactivation by reactive oxygen species (ROS). For instances, PTPN1 (PTP1B) is reversibly oxidized in response to epidermal growth factor receptor (EGFR) activation [4]. Similar modification of the catalytic cysteine residue has been shown for PTPN11 (SHP2) in PDGF signaling [5], PTPN1 and PTPN2 (TC-PTP) in insulin signaling [6], and PTPN6 (SHP1) in B cell receptor signaling [7,8]. In addition, SHP-1, SHP-2, and PTP1B are prone to oxidation by NO in the signaling of insulin or to ionization [9,10]. Therefore, transient burst of ROS and NO causing temporary inactivation of PTPs in response to PTK activation seems to be a general mechanism for maintaining high levels of tyrosine phosphorylation in the early phase of growth factor activation.

Malfunction of both PTKs and PTPs is involved in the development of some inherited and acquired human diseases [1,11,12]. For instance, PTP-1B has been linked to obesity and diabetes [13,14], PTP sigma to ulcerative colitis [15], and lymphoid-specific PTP (PTPN22) to autoimmune disorders [16,17]. Therefore, potent and specific PTP inhibitors can be used to study the role of PTPs in these diseases and be eventually developed into chemotherapeutic agents. Development of small molecule drugs targeting specific PTP is challenging because the PTP members are characterized by an exceptionally high degree of sequence conservation across their active sites [18,19]. Common approaches in developing novel small molecules directed to a particular enzyme include a traditional high-throughput screen using a chemical library and *in vitro* enzyme assays, synthesis of derivatives based on structure–activity relationship (SAR), and optimization of affinity and selectivity. Achieving target specificity may be the ultimate aim of drug development; however, it requires the knowledge of all targets of the drug. A previous report [20] estimated that a drug interacted on average with 6.3 targets. Thus, target identification of small molecule compounds seems to be the bottleneck of drug development [21].

**Figure 1:**
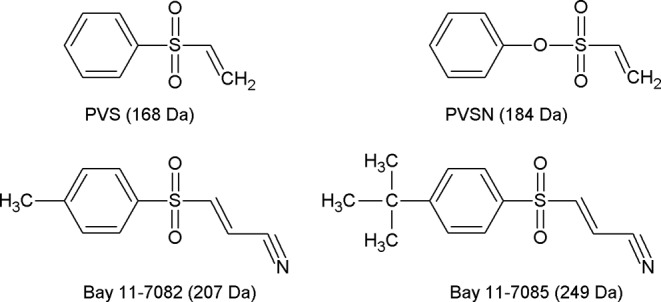
Structures of phenyl vinyl sulfone (PVS) and its derivatives

Phenyl vinyl sulfone (PVS) and phenyl vinyl sulfonate (PVSN) were characterized as a new class of mechanism-based PTP inhibitors [22]. These two compounds inactivate PTPs by mimicking the phosphotyrosine structure and providing a Michael addition acceptor for the active-site cysteine residue of PTPs (structures of PVS and related compounds illustrated in Figure 1). Based on these observations, we attempted to develop an antiserum against PVS and use the antiserum in the identification of PVS-tagged proteins through immunoprecipitation coupled with mass spectrometry analysis. Herein, using anti-PVS antiserum as an example, we have demonstrated the applications of antiserum against a covalent inhibitor in the identification of targets of inhibitors. PVSN and Bay 11-7082, structurally similar compounds to PVS, could inhibit the glutathione reductase activity *in vitro*. PVS, PVSN, and Bay 11-7082 could inhibit the protein arginine methyltransferase 1 (PRMT1) activity *in vitro*, and treatment of cells with PVSN, Bay 11-7082, or Bay 11-7085 caused the decline of the levels of protein asymmetric dimethylarginine catalyzed by PRMT1.

## Materials and methods

### Materials

High glucose Dulbecco’s Modified Eagle Medium, fetal bovine serum, Medium 199, OPTI-MEM, 0.25% trypsin-EDTA 1X (Gibco, Grand Island, NY); Immobilon Western chemiluminescent HRP substrate, C_18_ Zip-tip (Millipore, Billerica, MA); phenyl vinyl sulfone, phenyl vinyl sulfonate, ethyl vinyl sulfone, BVT 948, NSC 95397, Bay 11-7082, Bay 11-9085, AMI-1 (Santa Cruz Biotechnology, Santa Cruz, CA); trypsin (Promega, Madison, WI); recombinant PRMT1, PRMT1 assay kit (BPS Bioscience, San Diego, CA); histone H4 (New England BioLabs, Ipswich, MA); recombinant glutathione reductase, glutathione reductase activity colorimetric assay Kit (BioVision, Milpitas, CA); antibodies: IMPDH1 (PA5-27792, Thermo Fisher Scientific Inc,Waltham, MA); Anti-dimethyl-Arginine Antibody, asymmetric(ASYM24) (07-414), Anti-dimethyl-Histone H4 (Arg3) Asymmetric Antibody(07-213-I), Anti-phosphotyrosine Antibody, 4G10® Platinum (05-1050) (Millipore, Billerica, MA); (Cell Signaling Technology, Danvers, MA); PRMT1 (B-2, sc-166963), PTP1B (D-4, sc-133259), glutathione reductase (C-10, sc-133245), vimentin (H-84, sc-5565) (Santa Cruz Biotechnology, Santa Cruz, CA); peroxidase-conjugated secondary antibodies, Dylight™ 488, fluorescein (FITC)-conjugated secondary antibodies (Jackson ImmunoResearch Laboratories, West Grove, PA) were purchased from manufacturers indicated in parentheses.

### Cell culture

HeLa cells were obtained originally from American Type Culture Collection. Cells were cultured in Dulbecco’s Modified Eagle Medium (DMEM), high glucose medium containing 10% fetal bovine serum (FBS) within 5% CO_2_ atmosphere at 37°C.

### PVS, PVSN, or Bay 11-7082 tagging *in vitro*

HeLa cells were cultured to about 90% confluence on a 10-cm dish in DMEM with 10% FBS (5 × 10^6^ – 1 × 10^7^ cells). HeLa cells were lysed by repeated pipetting with 900 μl of 2% Triton X-100 in 20 mM Tris-HCl, pH 8.0. The lysate was centrifuged at 12000×***g*** for 10 min and the supernatant was aliquoted (200 μl) and treated with PVS, PVSN, or Bay 11-7082 of various concentrations at room temperature for 5 to 60 min. The stocks of 1000× PVS, 1000× PVSN, and 1000× Bay 11-7082 were prepared in DMSO. The reaction was stopped by adding 1,4-dithioerythreitol to a final concentration of 10 mM. The reaction solution was then mixed with equal volume of 2× SDS sample buffer. Ten microliters of sample solution was used for SDS gel electrophoresis and immunoblotting. The signal of GAPDH was used to adjust the amount of protein loading.

### PVS, PVSN, or Bay 11-7082 tagging *in cellulo*

HeLa cells were cultured to about 90% confluence on 10-cm dish in DMEM with 10% FBS (5 × 10^6^ – 1 × 10^7^ cells) for experiments. For PVS treatment, HeLa cells were refreshed with DMEM containing 10% FBS and various concentrations of PVS in the presence or absence of protein tyrosine phosphatase inhibitor such as pervanadate, BVT 948, or NSC 95397 for 5 to 60 min. For PVSN or Bay 11-7082 treatment, HeLa cells were washed with phosphate-buffered saline (PBS) and treated with PVSN or Bay 11-7082 in PBS for 5 to 60 min. The stock of 500× pervanadate was prepared in water. The stocks of 50 mM BVT 948, and 50 mM NSC 95397, 1000× PVS, 1000× PVSN, and 1000× Bay 11-7082 were prepared in DMSO solution. Each group contained the same volume of DMSO. After treatment, the cells were washed with PBS twice and lysed with 900 μl of Radioimmunoprecipitation Assay (RIPA) lysis buffer containing 10 mM 1,4-dithioerythritol. The solution was then mixed with equal volume of 2× SDS sample buffer. Ten microliters of sample solution was used for electrophoresis and immunoblotting. The signal of anti-GAPDH was used to adjust the amount of protein loading.

For the effects of PVS, PVSN, Bay 11-7082, Bay 11-7085, or AMI-1 on protein arginine methylation, HeLa cells were cultured to about 90% confluence on 10-cm dish in DMEM with 10% FBS (5 × 10^6^ – 1 × 10^7^ cells). After washing the HeLa cells with 10 ml of DMEM once, the cells were cultured for additional 1 or 3 h in 10 ml of DMEM containing PVS, PVSN, Bay 11-7082, Bay 11-7085, or AMI-1. The stocks of 1000× PVS, 1000× PVSN, 1000× Bay 11-7082, and 1000× Bay 11-7085 were prepared in DMSO solution, but 500× AMI-1 in ethanol. Each group contained the same volume of DMSO and ethanol. After wash with Tris-buffered saline (TBS) for three times, the cells were lysed with 900 μl of RIPA lysis buffer and centrifuged to pellet down the debris. The supernatant was then transferred to a new tube and mixed with equal volume of 2× SDS sample buffer. Ten microliters of sample solution was for electrophoresis and immunoblotting. The signal of anti-GAPDH was used to adjust the amount of protein loading.

### Immunoprecipitaton

After treatment with 1 mM PVS or PVSN for 30 min, the treated HeLa cells were lysed with an immunoprecipitation lysis buffer (50 mM MOPS, pH 7.2, 100 mM NaCl, 1 mM EDTA, 5% glycerol, and 1% NP-40) containing protease inhibitor cocktail. After centrifugation, the supernatant was added with anti-PVS and incubated at 4°C overnight. The immunocomplex was then pulled down by protein A-sepharose and washed by the immunoprecipitation lysis buffer three times. The captured proteins were eluted with SDS sample buffer.

### Preparation of anti-PVS antiserum

The antigen of PVS was prepared by coupling of the cysteine thiolate in reduced bovine serum albumin (BSA) to the terminal carbon of PVS under alkaline conditions. Two milliters of BSA at 2 mg/ml in PBS was reduced by 50 mM 1,4-dithioerythreitol at 37°C for 1 h. To this solution, 2 ml of 20% trichloroacetic acid was added. The mixture was mixed. Twenty millilters of ice-cold acetone was added and the mixture was mixed and kept at −20°C overnight. The resulting precipitate following low-speed centrifugation was dissolved in 2 ml of 0.1 M sodium carbonate buffer, pH 8.4, containing 4 mg PVS and the solution was incubated at 37°C for 4 h. The protein samples were buffer-exchanged into PBS by centrifugal concentration using an Amicon device with a cutoff of 10 kDa (Millipore) and then used for routine subcutaneous immunizations in rabbits. Following eight biweekly injections, whole blood was collected from the anesthetized animals 10 days after the final injection.

### In-gel digestion of HeLa cell lysate for MS analysis

After the SDS/PAGE fractionation, the gel band was cut into small pieces and reduced with 1,4-dithioerythreitol (50 mM) at 37°C for 1 h and alkylated with iodoacetamide (100 mM) at room temperature for 1 h. The gel pieces were destained repeatedly with 25 mM ammonium bicarbonate in 50% acetonitrile until became colorless. Gel slices were dehydrated with 100% acetonitrile for 5 min and vacuum-dried for 5 min. The followed enzymatic hydrolysis was carried out with trypsin at an enzyme-to-substrate ratio of 1/40 at 37°C for 16 h. The tryptic peptides were extracted twice with 50% acetonitrile containing 5% formic acid under moderate sonication for 10 min and dried completely under vacuum. The peptide mixtures were desalted by C18 Zip-tip and subjected to downstream MS analysis.

### Mass spectrometric analysis of proteins and data processing

The samples were reconstituted in 9% acetonitrile and 0.1% formic acid to give a volume of 4 µl, and loaded onto a C_18_ column of 75-μm × 250-mm (nanoACQUITY UPLC BEH130, Waters). The peptides mixtures were separated by online nanoflow liquid chromatography using nanoAcquity system (Waters) with a linear gradient of 5 to 50% acetonitrile (in 0.1% formic acid) in 95 min, followed by a sharp increase to 85% acetonitrile in 1 min and held for another 15 min at a constant flow rate of 300 nl min^−1^. Peptides were detected in an LTQ-OrbitrapVelos hybrid mass spectrometer (Thermo Scientific) using a data-dependent CID Top20 method in positive ionization mode. For each cycle, full-scan MS spectra (*m/z* 300–2000) were acquired in the Orbitrap at 60,000 resolution (at *m/z* 400) after accumulation to a target intensity value of 5 × 10^6^ ions in the linear ion trap. The 20 most intense ions with charge states ≥2 were sequentially isolated to a target value of 10,000 ions within a maximum injection time of 100 ms and fragmented in the high-pressure linear ion trap by low-energy CID with normalized collision energy of 35%. The resulting fragment ions were scanned out in the low-pressure ion trap at the normal scan rate and recorded with the secondary electron multipliers. Ion selection threshold was 500 counts for MS/MS, and the selected ions were excluded from further analysis for 30 s. An activation *q* = 0.25 and activation time of 10 ms were used. Standard mass spectrometric conditions for all experiments were: spray voltage, 1.8 kV; no sheath and auxiliary gas flow; heated capillary temperature, 200°C; predictive automatic gain control (AGC) enabled, and an S-lens RF level of 69%. All MS and MS/MS raw data were processed with Proteome Discoverer version 1.3 (Thermo Scientific), and the peptides were identified from the MS/MS data searched against the Swiss-Prot (540732 sequences entries) database using the Mascot search engine 2.3.02 (Matrix Science). Search criteria used were as follows: trypsin digestion; considered variable modifications of cysteine PVS-modification (+168.0245 Da), PVSN-modification (+184.01942 Da), glutamine deamidation (+0.98402 Da), methionine oxidation (+15.9949 Da), and cysteine carboxyamidomethylation (+57.0214 Da); up to three missed cleavages were allowed; and mass accuracy of 10 ppm for the parent ion and 0.6 Da for the fragment ions. The significant peptide hits defined as peptide score must be higher than Mascot significance threshold (*P* < 0.05) and therefore considered highly reliable, and that manual interpretation confirmed agreement between spectra and peptide sequence. After data acquisition, the individual MS/MS spectra within a single LC run were combined, smoothed, deisotoped using the MicromassProteinLynx™ Global Server (PGS) 2.2 and output as a single peak list (.pkl) file. The peak list files were used to query the Swiss-Prot database (SwissProt 54.1; 277883 sequences; 101975253 residues) using the MASCOT program (Version: 1.9.05) with the following parameters: peptide mass tolerance, 50 ppm; MS/MS ion mass tolerance, 0.25 Da; enzyme digestion was set to trypsin allow up to one missed cleavage; variable modifications considered were methionine oxidation and cysteine carboxyamidomethylation.

### *In vitro* glutathione reductase assay

The assay was carried out according to the protocol provided by BioVision Inc. (Catalog number K761-200). Recombinant glutathione reductase was first treated with NADPH and 10 or 50 μM PVS, PVSN or Bay11-7082 for 30 min at room temperature. In the control group, recombinant glutathione reductase was treated with NADPH and the same volume of DMSO used in the treatment of drugs in the assay buffer. Following the addition of oxidized glutathione, the decrease in absorbance at *A*_340_ was monitored over 10 min.

### *In vitro* protein arginine methyltransferase assay

The reaction mixture contained 100 ng recombinant PRMT1, 1 µg full-length recombinant Histone H4, 1 µM S-adenosylmethionine, various concentrations of PVS, PVSN, Bay 11-7082, or AMI-1 in a total volume of 100 µl in PBS, pH 7.4. The reaction was incubated at 37°C for 30 min and 10 µl of the reaction product was examined by SDS/PAGE and immunoblotting with anti-PVS and anti-H4R3me2a.

### LC–ESI–MS analysis for PVS, PVSN, or Bay 11-7082 adducts with cysteine

In 1 ml of solution containing 100 μM cysteine, 100 μM PVS, PVSN, or Bay 11-7082 in 20 mM NaHCO_3_, pH 8.4, reaction was held at 37°C for 60 min. The resulting reaction products were subjected directly to the LC–ESI–MS analyses. The LC–ESI–MS system consisted of an ultra-performance liquid chromatography system (Ultimate 3000 RSLC, Dionex) and an electrospray ionization (ESI) source of quadrupole time-of-flight (TOF) mass spectrometer (maXis HUR-QToF system, BrukerDaltonics). The samples were kept in an autosampler at 4°C. Separation was performed with reversed-phase liquid chromatography (RPLC) on a BEH C_18_ column (2.1 × 100 mm, Walters). The elution started from 99% mobile phase A (0.1% formic acid in ultrapure water) and 1% mobile phase B (0.1% formic acid in ACN), held at 1% B for 0.5 min, raised to 60% B in 6 min, further raised to 90% B in 0.5 min, held at 90% B for 1.5 min, and then lowered to 1% B in 0.5 min. The column was equilibrated by pumping 1% B for 4 min. The flow rate was set 0.4 ml/min with injection volume of 2 μl. LC–ESI–MS chromatogram were acquired under following conditions: capillary voltage of 4500 V in positive ion mode, dry temperature at 190°C dry gas flow maintained at 8 l/min, nebulizer gas at 1.4 bar, and acquisition range of *m/z* 100–1000.

### Other biochemical methods

Biochemical methods and immunological methods such as SDS/PAGE, immunoblotting, and immunofluorescence staining were essentially the same as described in our previous publication [23].

## Results

### Characterization of anti-PVS antiserum

**Figure 2:**
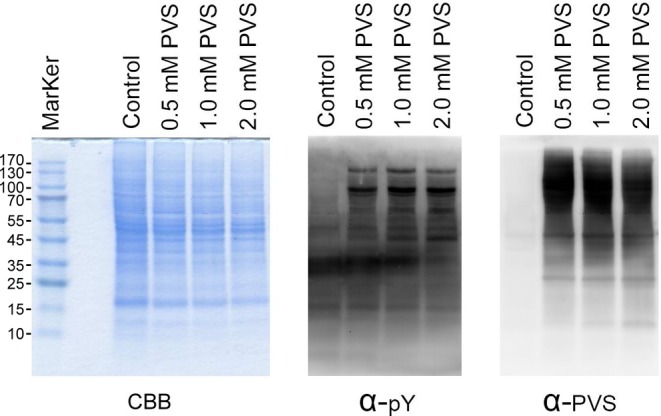
Phenyl vinyl sulfone (PVS) as a covalent protein tyrosine phosphatase (PTP) inhibitor HeLa cells were treated with various concentrations of PVS in PBS for 1 h, washed with PBS for three times, and lysed with RIPA lysis buffer containing 10 mM 1,4-dithioerythritol. The proteins in the lysate were examined by Coomassie blue G-250 staining (left panel), immunoblotting with anti-phosphotyrosine (middle panel), and anti-PVS (right panel) following SDS/PAGE and electrotransfer. The control groups were treated with the same volume of DMSO; α-PVS, anti-PVS; α-pY, anti-phosphotyrosine; CBB, Coomassie blue G-250.

Based on the observations that PVS and PVSN were PTP covalent inhibitors [22], we attempted to develop an antiserum against PVS and use the antiserum in the identification of PVS-tagged proteins through immunoblotting and immunoprecipitation. First, we confirmed the inhibitory activity of PVS on PTPs and tested the efficacy of our antiserum against PVS. HeLa cells were treated with various concentrations of PVS for 1 h and the cell lysate was examined by SDS/PAGE and immunoblotting using the antisera against phosphotyrosine and PVS (Figure 1). As expected, the levels of phosphotyrosine modification were highly elevated in cells following treatment of 0.5–2 mM PVS indicating that protein tyrosine phosphorylation in HeLa cells is constantly regulated by both PTKs and PTPs and PVS is a potent inhibitor of PTPs. Numerous proteins were covalently modified by PVS and revealed by the anti-PVS immunoblotting (Figure 2). Higher concentrations of PVS elicited higher levels of PVS modification, but with the same pattern. The antiserum showed great specificity as evidenced by the lack of signal in the control group. Specificity of the anti-PVS was further tested by the combined treatment of ethyl vinyl sulfone (EVS) and PVS. HeLa cells were pretreated with 10 μM to 1 mM EVS in PBS for 30 min and then treated with 100 μM PVS in PBS for 1 h. Pretreatment of EVS at 100 μM and 1 mM significantly attenuated PVS modification suggesting that the antiserum recognition relies heavily on the phenyl functional group (Supplementary Figure S1).

**Figure 3:**
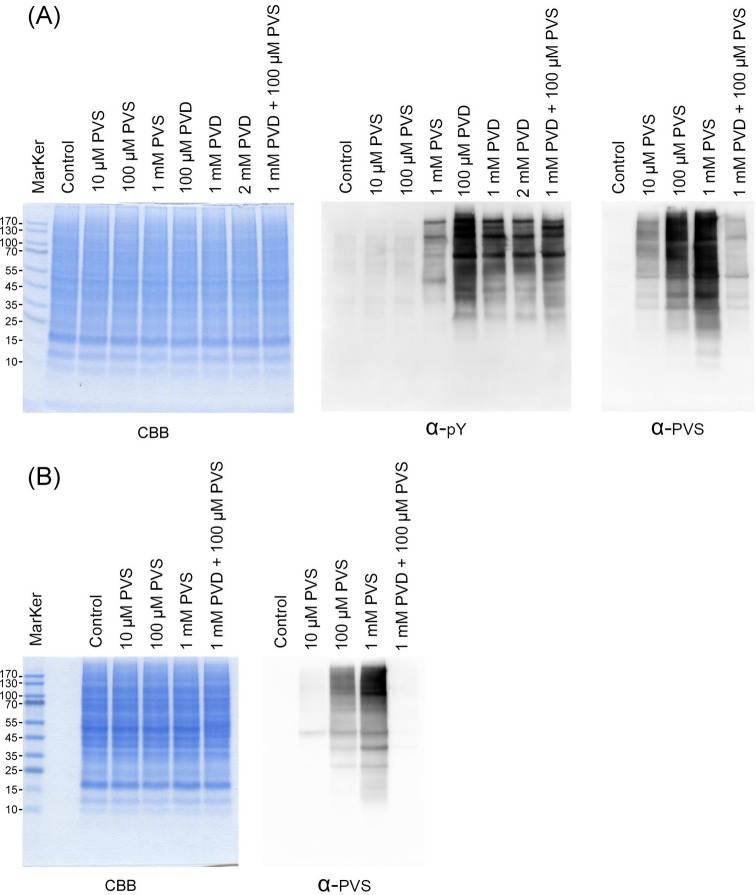
Competition of PVS labeling by a PTP inhibitor pervanadate (PVD) *in cellulo* (**A**) and *in vitro* (**B**) (**A**) HeLa cells were treated with 10 μM to 1 mM PVS in PBS for 1 h or with 0.1–2 mM PVD in PBS for 30 min. In the case of PVS and PVD combined treatment, HeLa cells were pretreated with PVD for 30 min and then treated with PVS in PBS for 1 h. The cells were then lysed with RIPA lysis buffer containing 10 mM 1,4-dithioerythritol. The proteins in the lysate were examined by Coomassie blue G-250 staining (left panel), immunoblotting with anti-phosphotyrosine (middle panel), and anti-PVS (right panel) following SDS/PAGE and electrotransfer. (**B**) HeLa cell lysates were treated with 10 μM to 1 mM PVS for 1 h. In the case of PVS and PVD combined treatment, HeLa cell lysates were pretreated with PVD for 30 min and then treated with PVS in PBS for 1 h. The reactions were then stopped by adding the same volume of RIPA buffer containing 10 mM 1,4-dithioerythritol. The proteins in the lysate were examined by Coomassie blue G-250 staining (left panel) anti-PVS (right panel) following SDS/PAGE and immunoblotting. The control groups were treated with the same volume of DMSO.

Pervanadate is an irreversible PTP inhibitor by oxidizing the active-site cysteine thiol of PTPs [24]. If both PVS and pervanadate target the same thiol group, pretreatment of pervanadate would hinder the tagging of PVS. Indeed, pretreatment of HeLa cells with 1 mM pervanadate for 30 min completely blocked the tagging of PVS (Figure 3A). Similarly, treatment of HeLa cell lysate with 1 mM pervanadate and 100 μM PVS together almost completely blocked the tagging of PVS (Figure 3B). Next, we tested the competition of BVT 948 and NSC 95397 on the tagging of PVS. BVT 948 [25] and NSC 95397 [26] are PTP and dual specificity phosphatase inhibitors respectively. Treatment of both drugs elicited increased amounts of tyrosine phosphorylation, albeit with different sensitivity and pattern (Supplementary Figure S2). NSC 95397 blocked PVS tagging better than BVT 948 both *in vitro* and *in cellulo*. Therefore, the data indicate that PVS forms covalent bond with the cysteine thiol groups of cellular proteins, which can be blocked by the pretreatment of known PTP inhibitors.

### Applications of anti-PVS antiserum

**Figure 4:**
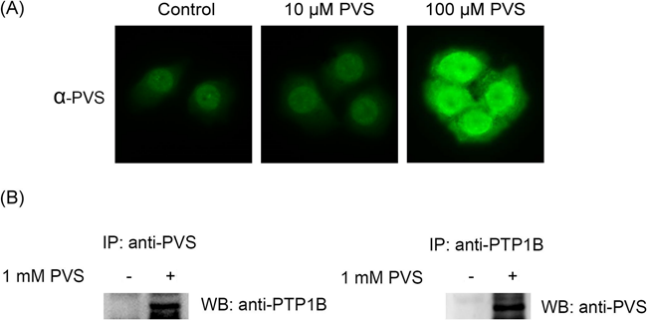
Applications of anti-PVS antiserum in immunofluorescence staining and immunoprecipitation experiments (**A**) HeLa cells were cultured on coverslips for at least 24 h and treated with 0–100 μM PVS in PBS for 1 h. The cells were then processed for routine immunofluorescence staining with anti-PVS and DyLight 488 labeled anti-guinea pig IgG secondary antibodies. (**B**) HeLa cells were treated with 1 mM PVS in PBS for 1 h and then lysed with a lysis buffer suitable for immunoprecipitation and then processed for routine immunoprecipitation experiments with anti-PVS or with anti-PTP 1B. The immunoprecipitates were then examined by SDS/PAGE and immunoblotting with anti-PTP1B or with anti-PVS. The control groups were treated with the same volume of DMSO.

Besides immunoblotting, we tested whether our anti-PVS antiserum is suitable for cell immunofluorescence staining and immunoprecipitation experiments. HeLa cells were treated with 10 and 100 μM PVS for 1 h and then processed with the standard procedures for immunofluorescence staining (Figure 4A). Immunoreactive signals were observed in the cytosol and nucleus and treatment with higher concentrations of PVS resulted in higher intensity of fluorescence signal. There was no fluorescence signal in the control cells. We then used anti-PVS antiserum to pull down PVS-tagged proteins from HeLa cells treated with 1 mM PVS for 1 h. Indeed, PTP1B was present in the PVS-tagged proteins as revealed by anti-PTP1B immunoblotting following anti-PVS immunoprecipitation (left panel, Figure 4B). Similarly, immunoprecipitation with anti-PTP1B antibody followed by immunoblotting with anti-PVS antiserum also confirmed the tagging of PTP1B with PVS (right panel, Figure 4B). Thus, anti-PVS antiserum can be used in the routine antibody-based assays such as immunoblotting, immunofluorescence staining, and immunoprecipitation.

**Figure 5:**
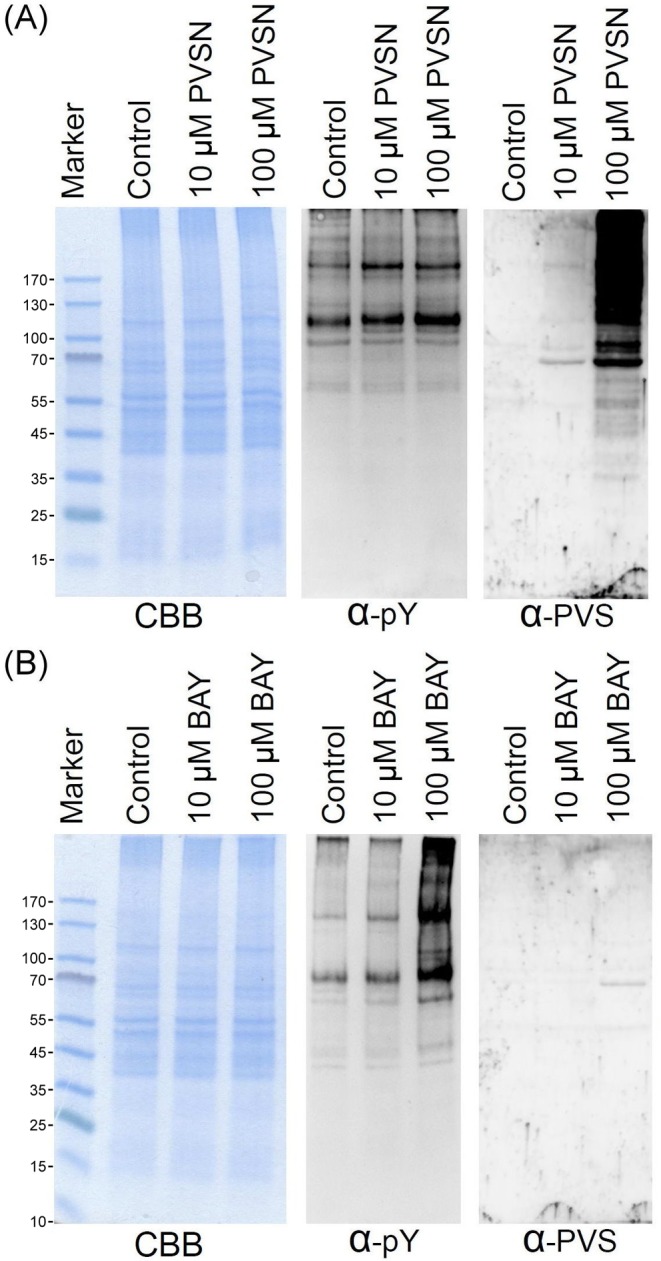
Use of anti-PVS in recognition of PVSN and Bay 11-7082 adducts HeLa cells were treated with various concentrations of PVSN (**A**) or Bay 11-7082 (**B**) in PBS for 5 min, washed with PBS for three times, and lysed with RIPA lysis buffer containing 10 mM 1,4-dithioerythritol. The proteins in the lysate were examined by Coomassie blue G-250 staining, immunoblotting with anti-phosphotyrosine, and anti-PVS following SDS/PAGE and electrotransfer. The control groups were treated with the same volume of DMSO; α-PVS, anti-PVS; α-pY, anti-phosphotyrosine; CBB, Coomassie blue G-250.

We next examined whether anti-PVS antiserum can be used in the detection of target proteins probed by PVS analogs such as PVSN and Bay 11-7082. Treatment of PVSN and Bay 11-7082 at concentrations of 10 and 100 μM for 5 min increased the levels of phosphotyrosine modification in HeLa cells, especially Bay 11-7082 at 100 μM (Figure 5) confirming their effects in PTP inhibition. PVSN-tagged proteins were abundantly recognized by anti-PVS antiserum (Figure 5A), but only few Bay 11-7082-tagged proteins were detected by the anti-PVS antiserum (Figure 5B). Therefore, anti-PVS antiserum can be readily used in the detection of proteins modified by PVS and PVSN.

### PVS-and PVSN-tagged proteins in HeLa cells

In order to find out the identity of PVS- or PVSN-tagged proteins, we used anti-PVS antiserum to pull down potential targets of PVS or PVSN in HeLa cells treated with 1 mM PVS or PVSN for 30 min. Cell lysates of control, PVS- or PVSN-treated HeLa cells were processed with immunoprecipitation by anti-PVS to obtain proteins potentially tagged by PVS or PVSN. In addition, tryptic peptides were prepared and used for immunoprecipitation by anti-PVS antiserum to reveal the modification sites. The false discovery rates were set to 0.01 for peptides, proteins and sites by target-decoy strategy to distinguish correct and incorrect identifications, with a cut-off adjusted *P* value ≤0.05. We assumed positive protein identification results when the Mascot scores of PVS- and PVSN-tagged proteins was higher than 50 and at least 2-fold higher than the corresponding control. The resulting data were summarized, and 183 candidates are listed in Supplementary Table S1. There are 103 target proteins tagged both by PVS and PVSN, 70 by PVS only, and 10 by PVSN only. It appears that PVS is less selective than PVSN in covalently tagging the target proteins. Surprisingly, only one PTP was found, the low molecular weight PTP, in the PVS-tagged proteins. One major concern of proteomics is that Mass spectrometer has a limited capacity in detecting low-abundance proteins (peptides) in samples with a wide range of relative abundance. Therefore, specific enrichment protocols are required for uncovering those low-abundance targets [27]. The data indicate that PVS and PVSN are reactive not only toward PTPs, but also toward other proteins, especially those with highly reactive cysteine residues or prone to oxidation [28,29]. It is generally believed that proteins with low p*K*_a_ thiols are susceptible to oxidation since thiolates are much stronger nucleophiles than thiol groups [30,31], and we marked proteins containing reactive cysteine or cysteine prone to oxidation with * or # respectively as shown in Supplementary Table S1 [28,29]. Modification sites of some PVS and PVSN targets were also determined with high confidence (Supplementary Tables S2 and S3). However, attempts to pull down Bay 11-7082-tagged proteins by anti-PVS were not successful**.**

**Figure 6:**
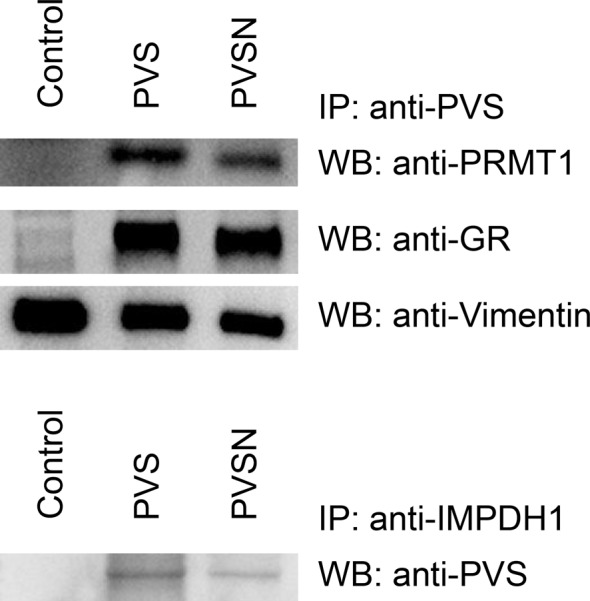
Confirmation of PVS and PVSN tagging by immunoprecipitation followed by immunoblotting HeLa cells were treated with 500 μM PVS or PVSN in PBS for 5 min and the lysate was subjected to anti-PVS immunoprecipitation. The immunoprecipitate was then probed with anti-protein arginine methyltransferase 1 (PRMT1), anti-glutathione reductase (GR), and anti-vimentin (upper panel). For inosine-5′-monophosphate dehydrogenase 1 (IMPDH1), the lysate was immunoprecipitated with anti-IMPDH1 and then the immunoprecipitate was probed with anti-PVS (lower panel). The control groups were treated with the same volume of DMSO.

We then chose protein arginine methyltransferase 1 (PRMT1), glutathione reductase, vimentin, and inosine-5′-monophosphate dehydrogenase 1 (IMPDH1) for further study due to our interest and their relatively high scores in the proteomics data. Immunoprecipitation was carried out with anti-PVS in cell lysate prepared from HeLa cells treated with PVS or PVSN and the immunoprecipitate was subjected to immunoblotting (Figure 6). For IMPDH1, cell lysate was immunoprecipitated with anti-IMPDH1 followed by immunoblotting with anti-PVS. The results confirmed that PRMT1, glutathione reductase, and IMPDH1 were indeed tagged by PVS or PVSN in cells treated with PVS or PVSN. Whether vimentin was tagged by PVS or PVSN was inconclusive since its signal was also present in the control group. However, modification site was identified by Mass spectrometric analysis suggesting that vimentin is indeed tagged by PVS or PVSN. Therefore, the data indicate that both PVS and PVSN are not specific for PTPs. They form covalent adducts with a wide variety of proteins other than PTPs.

### PVS, PVSN, and Bay 11-7082 as PRMT1 inhibitors *in vitro*

**Figure 7:**
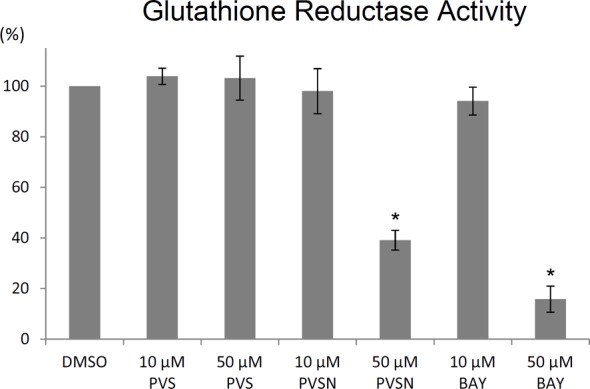
Effects of PVS, PVSN, and Bay 11-7082 on the *in vitro* enzyme activity of glutathione reductase Recombinant glutathione reductase was first treated with NADPH and 10 or 50 μM PVS, PVSN, or Bay 11-7082 for 1 h at room temperature. In the control group, recombinant glutathione reductase was treated with NADPH and the same volume of DMSO used in the treatment of drugs in the assay buffer. Following the addition of oxidized glutathione, the decrease in absorbance at *A*_340_ was monitored over 10 min. Results were presented as mean of three independent experiments plus and minus standard deviation (compared with DMSO, *P<0.001.)

Based on the proteomic results, we first examined whether PVS, PVSN, and Bay 11-7082 affected the activity of glutathione reductase (Figure 7). All compounds were ineffective in inhibiting glutathione reductase at 10 µM. However, PVSN and Bay 11-7082 at 50 µM inhibited the activity of glutathione reductase by 61% and 74% respectively. It is highly possible that PVSN and Bay 11-7082 inhibit glutathione reductase at high concentrations by tagging one of the cysteine residues (Cys58) involved in the catalytic reduction of oxidized glutathione [32] (Supplementary Table S3). A previous report also supports this result by showing that Bay 11-7082 inhibited GR activity in erythrocytes [33].

**Figure 8:**
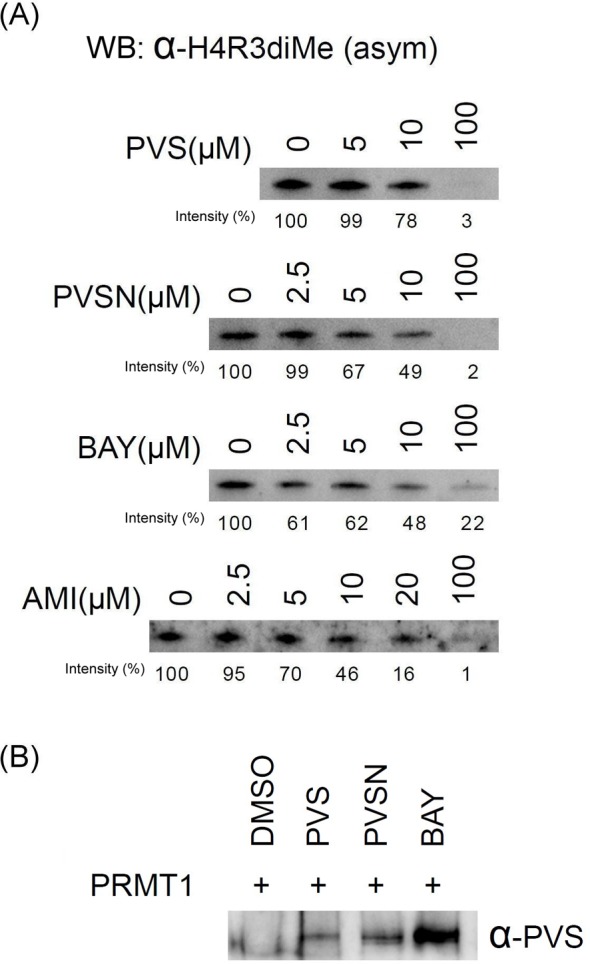
Effects of PVS, PVSN, Bay 11-7082, and AMI-1 on the *in vitro* enzyme activity of PRMT1 (**A**) and anti-PVS detection of PVS, PVSN, and Bay 11-7082 tagging of PRMT1 (**B**) The reaction mixture contained 100 ng of recombinant PRMT1, 1 µg of full-length recombinant Histone H4, 1 µM S-adenosylmethionine, various concentrations of PVS, PVSN, Bay 11-7082, or AMI-1 (AMI) in a total volume of 100 µl in PBS, pH 7.4. DMSO was used in the control. The reaction was incubated at 37°C for 30 min and 10 µl of the reaction product was examined by SDS/PAGE and immunoblotting with anti-H4R3me2a for the recognition of Histone H4 asymmetric dimethylation at Arg3 (**A**) and anti-PVS (**B**). The 0 µM groups were treated with the same volume of DMSO.

**Figure 9:**
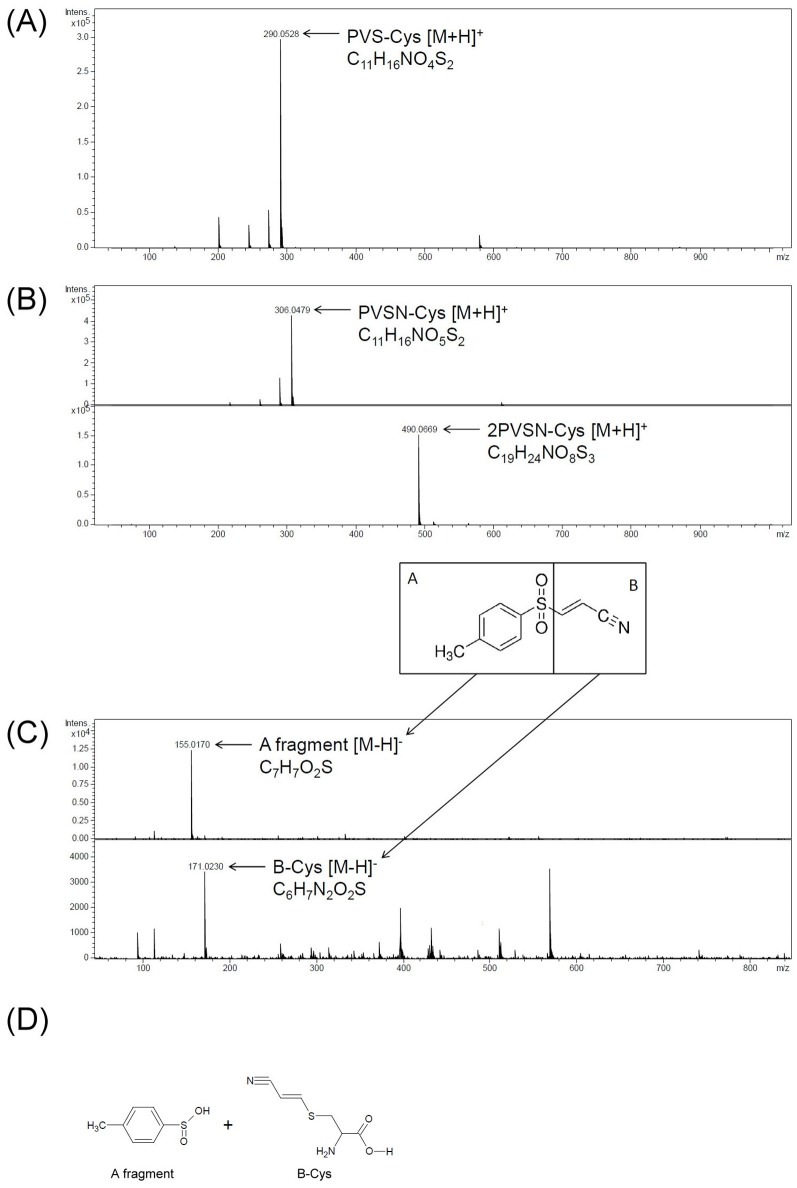
LC–MS analysis of reaction products of PVS, PVSN, and Bay 11-7082 with cysteine In 1 ml of solution containing 100 μM cysteine, 100 μM PVS, PVSN, or Bay 11-7082, 20 mM NaHCO_3_ pH 8.4, the reaction was held at 37°C for 1 h. The reaction products were then analyzed by LC–ESI–MS. (**A**) The calculated molecular mass of PVS-cysteine adduct is 289 Da, and the calculated *m/z* [M + H]^+^ is 290 Da/e. The extracted ions of *m/z* 290.0528 Da/e represented PVS-cysteine adduct. (**B**) The calculated molecular mass of PVSN-cysteine adduct is 305 Da, and the calculated *m/z* [M+H]^+^ is 306 Da/e. The extracted ions of *m/z* 306.0479 Da/e represented PVSN-cysteine adduct. Extracted ions of *m/z* 490.0669 Da/e represented 2PVSN-cysteine because the calculated molecular mass of 2PVSN-cysteine adduct is 489 Da, and the calculated *m/z* [M + H]^+^ is 490 Da/e. (**C**) The extracted ions of *m/z* 155.0170 and 171.0230 Da/e represented Bay 11-7082 A fragment and B fragment-cysteine adduct respectively. The calculated molecular mass of A fragment-cysteine adduct is 156 Da (172 Da for B fragment-cysteine adduct), and the calculated m/z [M − H]^−^ is 155 Da/e (171 Da/e for B fragment-cysteine adduct). (**D**) The structures of Bay 11-7082 A fragment and B fragment-cysteine adduct were shown. Ions originating from original molecules by addition of a proton [M + H]^+^ or abstraction of a proton [M − H]^−^ were observed in positive or negative ion mode respectively.

Protein arginine methylation catalyzed by PRMTs results in the addition of methyl groups to the nitrogen atoms of the arginine side chains in the forms of monomethylated (N^G^-monomethylarginine), symmetrically dimethylated (N^G^,N′^G^-dimethylarginine), and asymmetrically dimethylated arginine (N^G^,N^G^-dimethylarginine; ADMA). Multiple cellular processes, including chromatin structure, signal transduction, transcriptional regulation, RNA metabolism, and DNA damage repair, are regulated by protein arginine methylation [34]. PRMT1 is responsible for most ADMA formation in cells [35] such as histone H4 arginine 3 (H4R3) ADMA [36,37]. The protein identification and modification site identification results suggest that PVS or PVSN may serve as an inhibitor of PRMT1. Especially, one highly reactive cysteine residue was tagged by iodoacetamide [28] and PVS (Supplementary Table S2), whose tagging may lead to inactivation of PRMT1 [28]. *In vitro* PRMT1 activity assay using recombinant PRMT1, histone H4, and S-adenosylmethionine in the presence of PVS, PVSN, or Bay 11-7082 was conducted (Figure 8A). Bay 11-7082 at 2.5 µM, PVSN at 5 µM, and PVS at 10 µM caused significant inhibition of methylation of histone H4. AMI-1, a well-known inhibitor of PRMT1 [38], was used for comparison. The results indicate that PVSN and Bay 11-7082 are close to AMI-1 in PRMT1 inhibitor potency using histone H4 as a substrate. Based on the results in Figure 8A, we calculated the IC_50_ of PVS, PVSN, Bay 11-7082, and AMI-1 as 23.32, 10.38, 10.72, and 10.4 µM respectively by direct curve-fitting logistic regression analysis using the data of 5, 10, and 100 µM treatments. Three-point IC_50_ curves may provide an estimation, but certainly not an accurate calculation (data is not shown). Interestingly, adduct of Bay 11-7082 with PRMT1 can be recognized by anti-PVS (Figure 8B). Our previous data showed that only few Bay 11-7082-tagged proteins were detected by the anti-PVS antiserum (Figure 5). We then examined the reactivity of PVS, PVSN, and Bay 11-7082 with free cysteine in mild alkaline solution and examined the reaction products with LC–ESI–MS analysis (Figure 9). PVS and PVSN readily formed adducts with cysteine through the expected Michael addition reaction (Figure 9A,B, and Supplementary Figure S3), and we noticed an additional trace products appearing in the PVSN-cysteine reaction (Figure 9B and Supplementary Figure S4). However, the major reaction product of Bay 11-7082 with cysteine was obtained through substitution at the C3 position, but not by Michael addition (Figure 9C,D, and Supplementary Figure S3). The data indicate that PVS, PVSN, and Bay 11-7082 are all inhibitors of PRMT1 *in vitro* through covalent modification of the enzyme and these compounds can serve as the lead compound of PRMT1 inhibitor development.

### Bay 11-7082 as a PRMT1 inhibitor *in cellulo*

**Figure 10:**
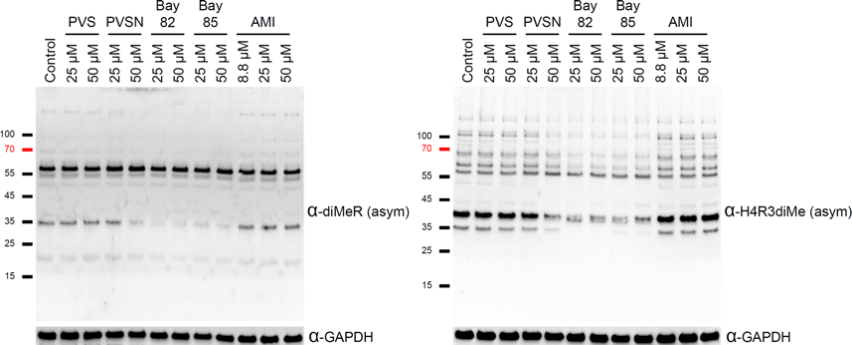
Effects of PVS, PVSN, Bay 11-7082, Bay 11-7085, and AMI-1 on the signals of asymmetric dimethylarginine in HeLa Cells HeLa cells were treated with 25 or 50 μM compounds in DMEM without FBS for 1 h. After washed with TBS for three times, the cells were then lysed and examined by SDS/PAGE and immunoblotting with anti-H4R3me2a antibody (α-H4R3diMe, asym) and with anti-dimethylarginine antibody (asymmetric) (α-diMeR, asym). The control groups were treated with the same volume of DMSO.

Since Bay 11-7082 inhibited PRMT1 activity in an *in vitro* assay, we then tested the inhibitory activity of this compound in cultured HeLa cells. The levels of ADMA are regulated by both methylation and demethylation. Protein arginine dimethylation levels do not seem as dynamic as protein tyrosine phosphorylation levels since ADMA levels decreased only by 50% 7 days after induction of PRMT1 knockout in mouse embryonic fibroblast [39]. Some proteins of ADMA persisted several days in the absence of PRMT1. Interestingly, treatment of Bay 11-7082 in the cell culture condition for 3 h led to decline of levels in asymmetric dimethylarginine (ADMA) of 25 and 35 kDa (left panel, Supplementary Figure S5). We also used histone H4R3 asymmetric dimethylation antibody as one kind of pan ADMA antibody (right panel, Supplementary Figure S5). However, we noticed that the ADMA signals of one or two proteins increased in this experiment possibly due to the compensation activity caused by other PRMT family proteins [39] or disturbance of cell cycle progression affected by other target proteins of Bay 11-7082 [40]. It is of interest to note that phenylsulfonyl structure present in Bay 11-7082 is also found in a PRMT1 inhibitor, C-7280948 [41] (Supplementary Figure S6), indicating the Bay 11-7082 could be a good lead compound for developing PRMT1 inhibitors. Therefore, we further compared the effects of PVS, PVSN, Bay 11-7082, Bay 11-7085, and AMI-1 under the cell culture conditions. We included another Bay 11-7082 analog, Bay 11-7085 (Figure 1) for comparison [42,43]. Surprisingly, PVS at 50 μM did not change the levels of protein ADMA in cell culture (Figure 10), but PVSN at 50 μM decreased the levels of protein ADMA slightly (Figure 10). Treatments of Bay 11-7082 or Bay 11-7085 at concentration higher than 25 μM for 1 h led to decline of signals of ADMA. By the short-term treatment, AMI-1 at 50 μM had little effect on the general protein ADMA (Figure 10) although 7-day treatment of AMI-1 in HeLa cells led to decrease in arginine methylation of Npl3 protein [38]. The data indicate that PVSN, Bay 11-7082, and Bay 11-7085 are effective *in cellulo* in decreasing the levels of protein ADMA possible due to the inhibition of PRMT1.

## Discussion

We have developed a novel method to unravel potential targets of covalent inhibitors using an antiserum against PVS as an example. Target identification through immunoprecipitation and mass spectrometric analysis reveals potential targets of PVS and PVSN. Among the targets, PRMT1, IMPDH1, vimentin, and glutathione reductase have been further confirmed by immunoprecipitation followed by immunoblotting. In addition, the inhibition of PRMT1 activity by PVS, PVSN, and Bay 11-7082 was confirmed by an *in vitro* assay. The effects of PVSN, Bay 11-7082, and Bay 11-7085 on protein arginine dimethylation were also confirmed in cultured cells. All of the above compounds contain phenylsulfonyl moiety in structure which may be required for PRMT1 binding. Furthermore, inhibitory potency against PRMT1 increases on increasing structural complexity from PVS to Bay 11-7082. It appears that PVS and PVSN are non-specific thiol-targeting agents like iodoacetamide rendering them less potent *in cellulo*. Therefore, this novel approach can be used in the identification of potential targets of covalent drugs or activity-based protein profiling probes.

Targeted covalent drugs have recently gained more attention, particularly protein kinase inhibitors [44–46]. These drugs target a noncatalytic nucleophile that is unique for each target protein in contrast with the catalytic nucleophile in mechanism-based or suicide inhibitors. The risk of toxicity associated with covalent drugs can be largely circumvented by optimization of non-covalent interactions and optimal positioning of the electrophile relative to the nucleophile on the target. Inspired by the satisfactory outcome from the clinical trials of afatinib and ibrutinib [47–51], more targeted covalent drugs will be designed and synthesized. Our approach using antibodies against covalent drugs can provide an effective method in unraveling potential targets of targeted covalent drugs in the process of drug development.

Activity-based protein profiling (ABPP) utilizes chemical probes targeting covalently the active site of one enzyme or enzymes of a particular clan. Within the activity-based probes, a reporter group or a tag is usually designed to provide opportunities for visualization or capture of target proteins of interest [52,53]. One important feature of ABPP is that the probe registers only levels of the active enzyme instead of total protein revealed by immunoblotting. However, reporter groups containing fluorophores or biotin are relatively bulky that may hinder the interaction of the reactive group with the active site of target proteins. In addition, biotin moiety and most fluorophore structures are not cell-permeable rendering many activity-based probes incompatible for *in cellulo* or *in vivo* studies [54–56]. However, a terminal alkyne tag which is small and chemically inert within cells provides a functional group for bio-orthogonal click chemistry with high efficiency. Importantly, these alkyne-containing probes are suitable for *in cellulo* protein labeling [57,58]. Activity-based probes for protein tyrosine phosphatase have been developed [59,60]. Although these α-bromobenzylphosphonate-based probes are more specific for PTPs than 4-fluromethylaryl phosphate derivatives, they are not readily permeable to the biological membranes. On the other hand, PVSN and PVS are mechanism-based probes for the PTPs and azide-tagged PVSN probe is membrane permeable [22] providing an activity-based probe for PTPs. However, the exact targets of PVS and PVSN have not been examined in details until this communication. The present study indicates that antibodies against activity-based probes can be an alternative in detection of potential targets.

Bay 11-7082 [(E)-3-(4-Methylphenylsulfonyl)-2-propenenitrile] was originally described as an inhibitor of the activation of the transcription factor NF-κB [61]. However, the presence of the vinyl group in Bay 11-7082 suggests that it may serve as a Michael acceptor and function as an irreversible inhibitor of the PTPs [62]. Indeed, Bay 11-7082 treatment caused an increase in protein tyrosine phosphorylation and then prohibited LPS-stimulated phosphotyrosine levels in cultured RAW 264 macrophage [62]. In addition, mass spectrometry analysis showed the presence of predicted Bay 11-7082 adduct of PTP1B possibly occurring through Michael addition at the C2 position [62]. Unexpectedly, our anti-PVS antiserum detected few signals in cells treated with Bay 11-7082 (Figure 5B), which might not be due to structural difference between PVS and Bay 11-7082. We then studied the reaction mechanism by incubating free cysteine with Bay 11-7082 and analyzing the reaction products by LC–ESI–MS (Figure 9). The data indicate that the reaction occurs at the C3 carbon atom of Bay 11-7082 with the elimination of 4-methylbenzene-sulfinic acid, thus confirming the data published by Cohen and coworkers [63]. However, anti-PVS did recognize the conjugated adduct of Bay 11-7082 with PRMT1 (Figure 8B) suggesting a reaction via Michael addition at the C2 position. Therefore, target proteins may react with Bay 11-7082 through two distinct reaction mechanisms; either addition at C2 or substitution at C3 position due to the presence of two electron-withdrawing functional groups in Bay 11-7082. Reactions with cysteine molecule favor the substitution at C3 position while reactions with cysteine residues of proteins may undergo either reaction path [62,63] (Supplementary Figure S3).

In conclusion, our antibody-based target identification method can be used in fishing out the potential targets of covalent drugs. In particular, synthesis of a typical activity-based probe needs detailed SAR studies to know the desired sites of attachment of linker and handle. This creates difficulties in the synthesis of the probes needed. Therefore, our approach is most welcome when synthesis of an activity-based probe from a known covalent inhibitor, such as electrophilic natural products [64], is difficult to achieve.

## Supporting information

**supplementary Figures:** 

**Supplementary Table S1.** Identified PVS- and PVSN-tagged proteins in HeLa cells

**Supplementary Table S2.** Identified Modification Sites of PVS-tagged Proteins

**Supplementary Table S3.** Identified Modification Sites of PVSN-tagged Proteins

## Abbreviations

BAY: Bay 11-7082
BSA: bovine serum albumin
EGFR: epidermal growth factor receptor
EVS: ethyl vinyl sulfone
FBS: fetal bovine serum
GR: glutathione reductase
IMPDH1: inosine-5′-monophosphate dehydrogenase 1
PBS: phosphate-buffered saline
PRMT1: protein arginine methyltransferase 1
PTK: protein tyrosine kinase
PTP: protein tyrosine phosphatase
PVD: pervanadate
PVS: phenyl vinyl sulfone
PVSN: phenyl vinyl sulfonate
RIPA: radioimmunoprecipitation assay
ROS: reactive oxygen species
TBS: Tris-buffered saline
