# Supplementary material for: Target identification reveals protein arginine methyltransferase 1 is a potential target of phenyl vinyl sulfone and its derivatives
Source: Biosci Rep. 2018 Apr 20;38(2):BSR20171717. doi: 10.1042/BSR20171717 (PMC5968187; doi:10.1042/BSR20171717)
Supplement: Supplementary file 1 [file bsr20171717_Supp1.pdf]

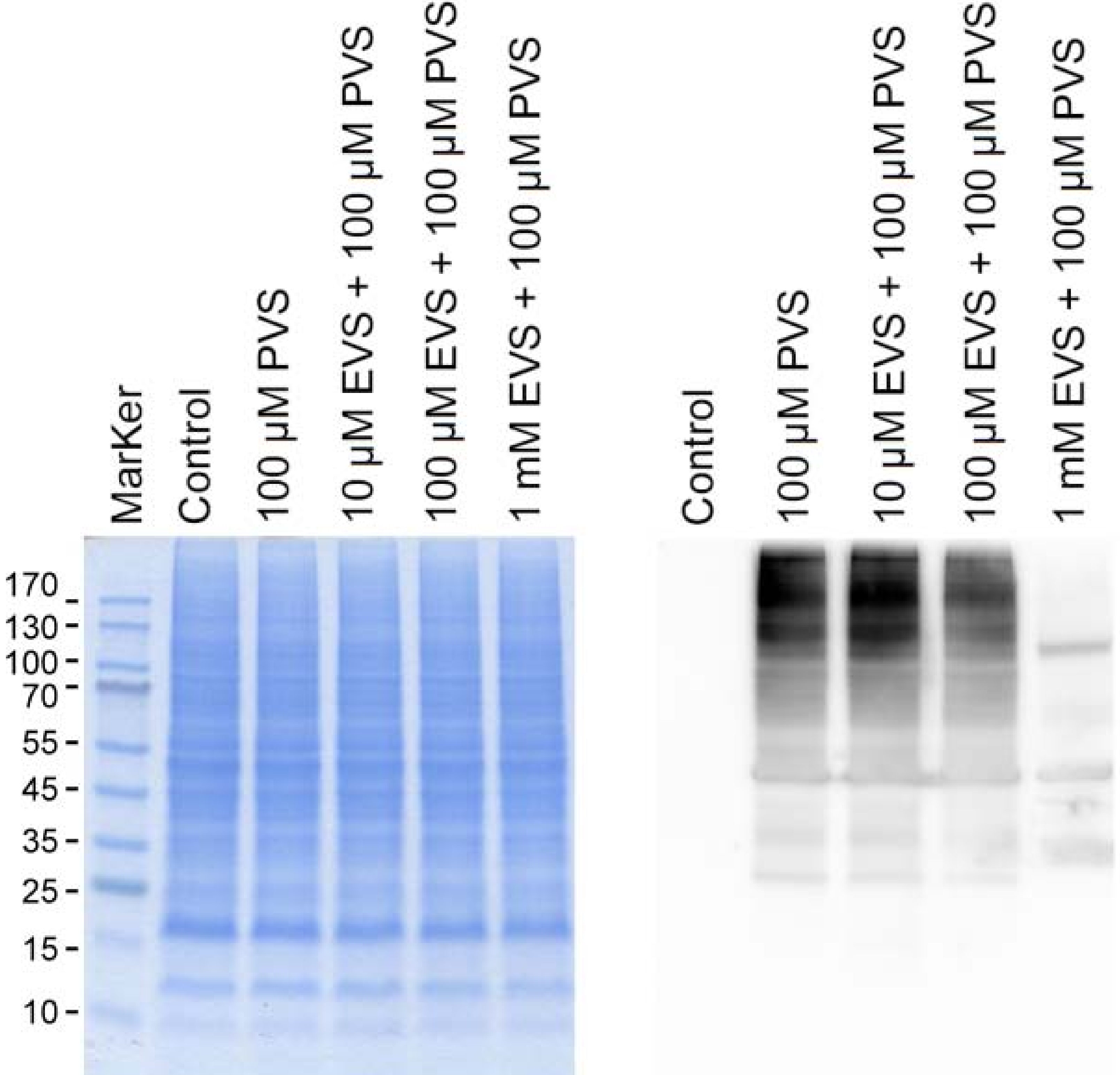

MarKer
Control
100 μM PVS
10 μM EVS + 100 μM PVS
100 μM EVS + 100 μM PVS
1 mM EVS + 100 μM PVS
Control
100 μM PVS
10 μM EVS + 100 μM PVS
100 μM EVS + 100 μM PVS
1 mM EVS + 100 μM PVS
170
130
100
70
55
45
35
25
15
10
CBB
α-PVS

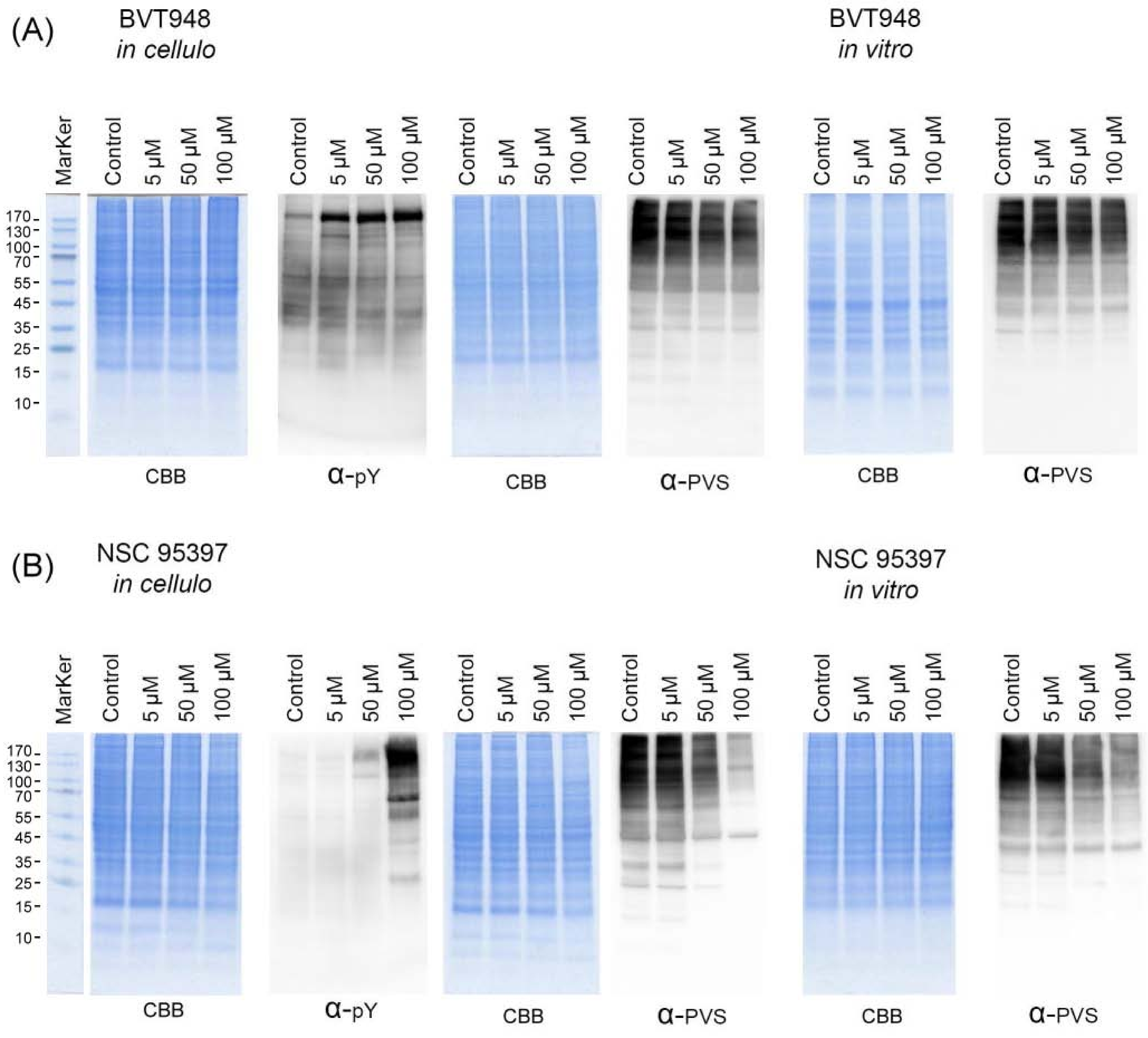

(A)
BVT948
in cellulo
BVT948
in vitro
MarKer
Control
5 μM
50 μM
100 μM
170
130
100
70
55
45
35
25
15
10
CBB
α-pY
CBB
α-PVS
CBB
α-PVS
(B)
NSC 95397
in cellulo
NSC 95397
in vitro
CBB
α-pY
CBB
α-PVS
CBB
α-PVS

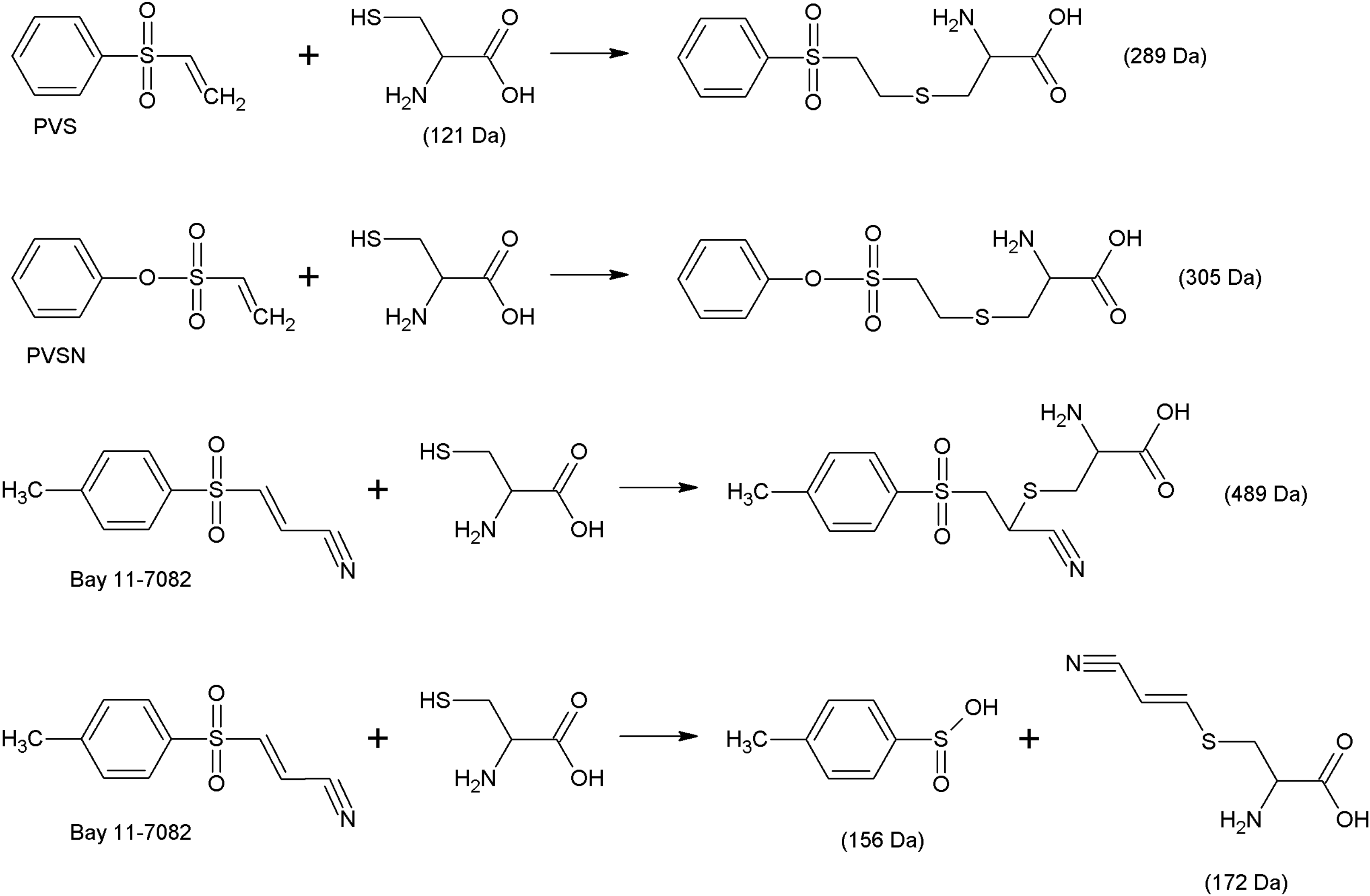
PVS
(121 Da)
(289 Da)
PVSN
(305 Da)
Bay 11-7082
(489 Da)
Bay 11-7082
(156 Da)
(172 Da)

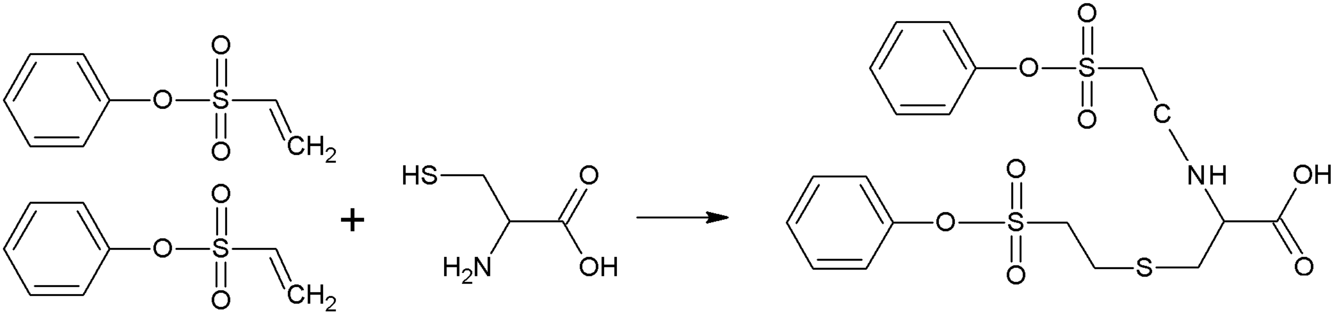

PVSN

(489 Da)

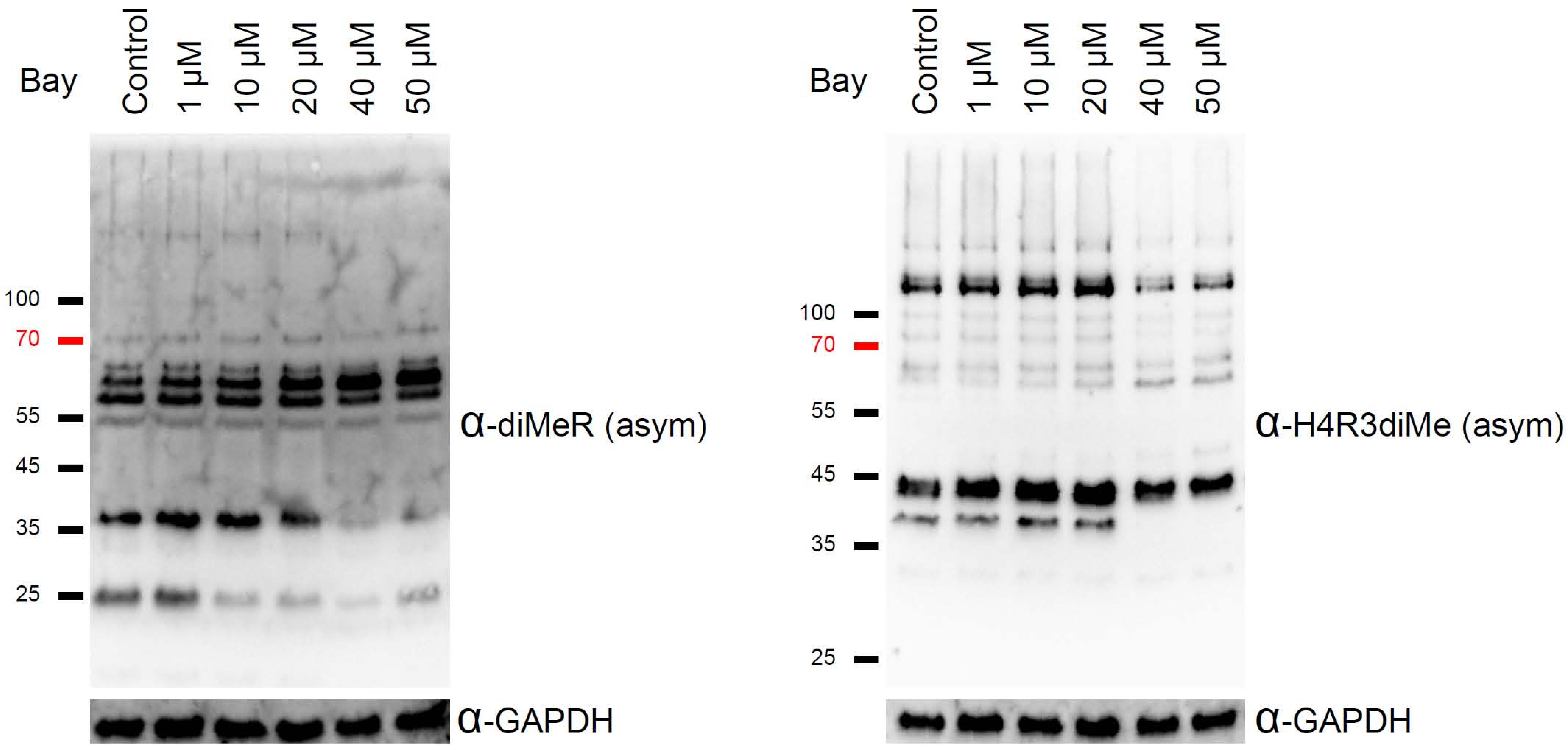

Bay
Control
1 μM
10 μM
20 μM
40 μM
50 μM
100
70
55
45
35
25
α-diMeR (asym)
α-GAPDH
Bay
Control
1 μM
10 μM
20 μM
40 μM
50 μM
100
70
55
45
35
25
α-H4R3diMe (asym)
α-GAPDH

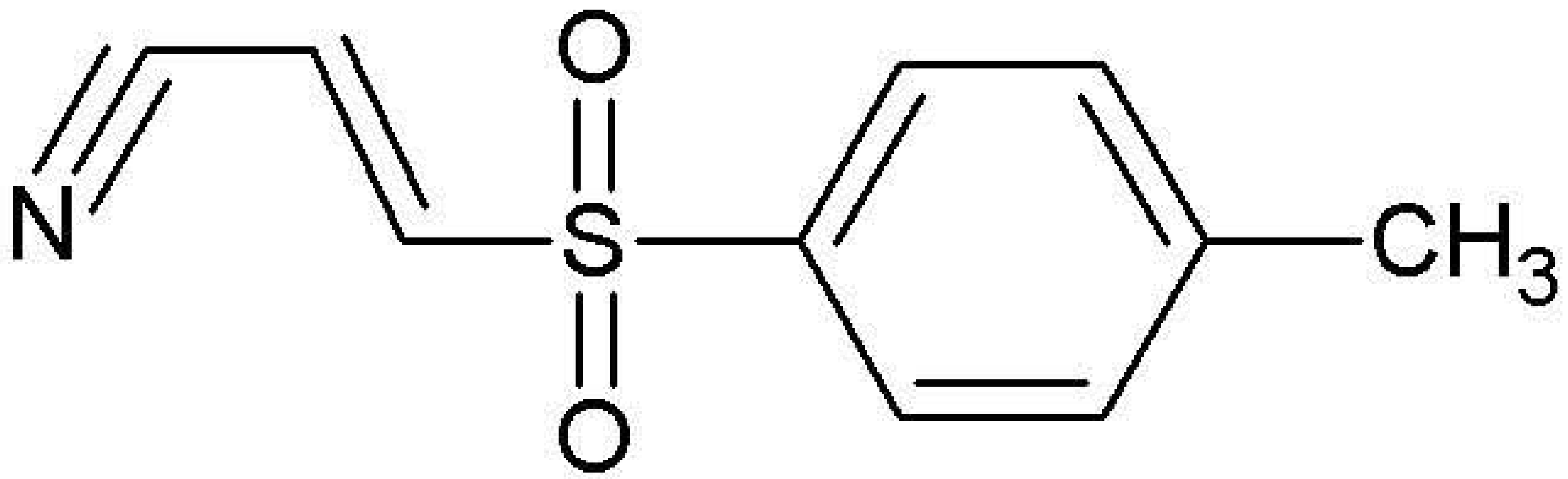

Bay 11-7082

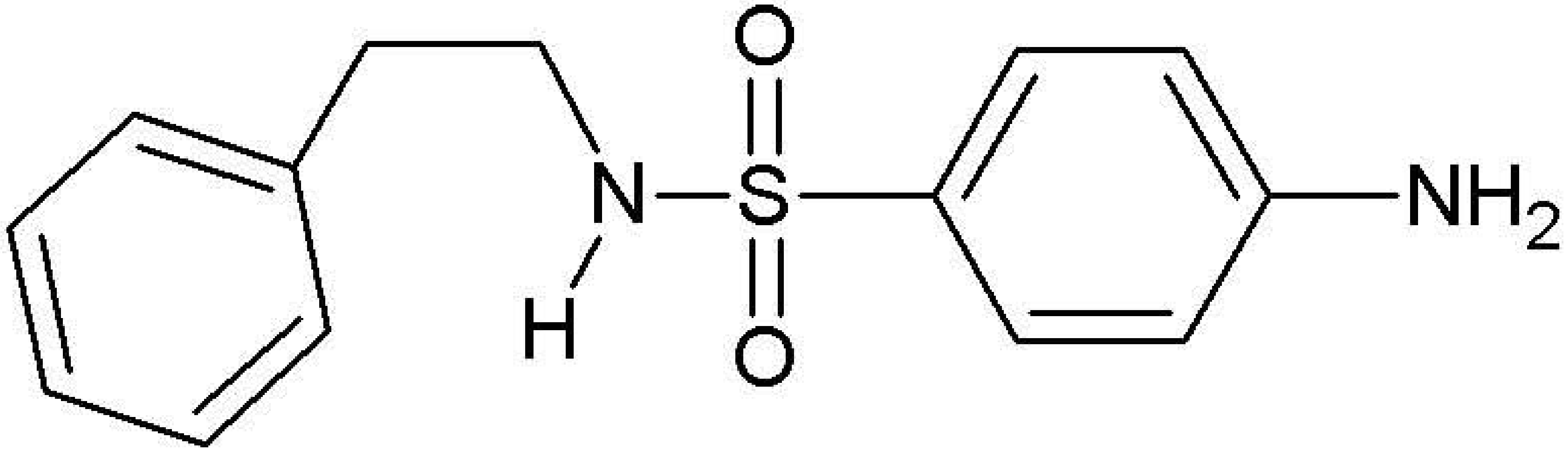

C-7280948

Supplementary Table S1.

Identified PVS- and PVSN-tagged proteins in HeLa cells

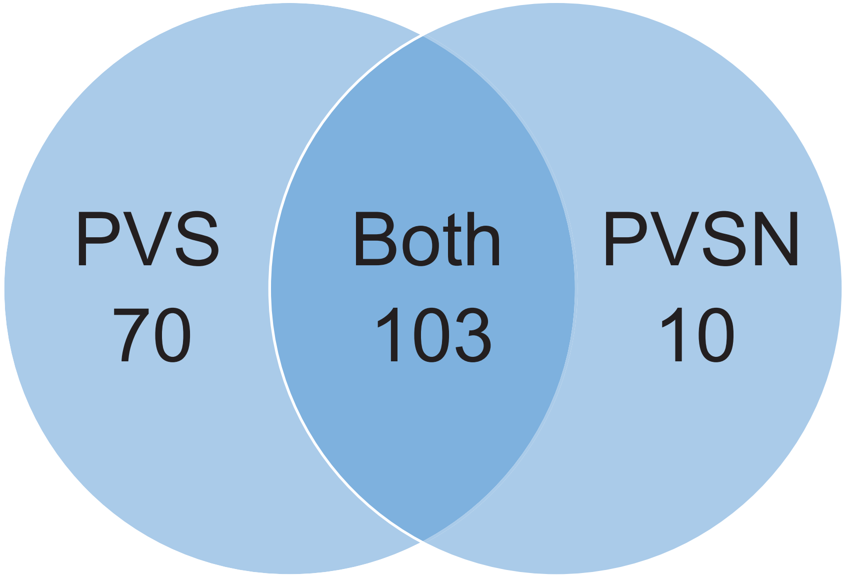

Supplementary Table S1. Identified PVS- and PVSN-tagged proteins in HeLa cells

Proteins tagged by	Accession #	Protein Species	PVS IP			PVSN IP			MW (kDa)
			Score	Peptides #	Coverage (%)	Score	Peptides #	Coverage (%)	
PVS	Q13501	Sequestosome-1	711.21	6	23.41				47.7
PVS	P11413	Glucose-6-phosphate 1-dehydrogenase*#	490.74	15	32.04				59.2
PVS	P33316	Deoxyuridine 5'-triphosphate nucleotidohydrolase, mitochondrial*	447.06	5	25				26.5
PVS	Q9UHR4	Brain-specific angiogenesis inhibitor 1-associated protein 2-like protein 1	401.49	4	9.2				56.8
PVS	P62851	40S ribosomal protein S25	289.03	3	24				13.7
PVS	A4D1P6	WD repeat-containing protein 91	275.43	7	12.85				83.3
PVS	Q06210	Glutamine--fructose-6-phosphate aminotransferase [isomerizing] 1	262.46	4	7.01				78.8
PVS	P62249	40S ribosomal protein S16	257.99	6	34.93				16.4
PVS	Q96QR8	Transcriptional activator protein Pur-beta	231.47	8	19.87				33.2
PVS	P62266	40S ribosomal protein S23*	222.32	1	7.69				15.8
PVS	P83731	60S ribosomal protein L24	217.39	3	19.11				17.8
PVS	Q53H96	Pyrroline-5-carboxylate reductase 3	214.55	4	`5.69				28.6
PVS	P06899	Histone H2B type 1-J	208.72	3	26.98				13.9
PVS	Q16881	Thioredoxin reductase 1, cytoplasmic*	203.08	4	6.16				70.9
PVS	P26599	Polypyrimidine tract-binding protein 1	202.02	4	8.29				57.2
PVS	Q9Y5X1	Sorting nexin-9	196.85	4	8.07				66.5
PVS	P62847	40S ribosomal protein S24	190.06	2	20.3				15.4
PVS	P62750	60S ribosomal protein L23a	187.78	4	20.51				17.7
PVS	P62854	40S ribosomal protein S26	186.55	3	33.91				13
PVS	Q9HB71	Calcyclin-binding protein	184.63	6	27.19				26.2
PVS	P00558	Phosphoglycerate kinase 1*#	182.51	3	10.79				44.6
PVS	P39019	40S ribosomal protein S19	181.89	7	32.41				16.1
PVS	O76094	Signal recognition particle 72 kDa protein	180.42	2	4.02				74.6
PVS	P31151	Protein S100-A7	170.15	3	23.76				11.5
PVS	Q9UHD2	Serine/threonine-protein kinase TBK1	165.83	3	5.76				83.6
PVS	P84103	Serine/arginine-rich splicing factor 3	164.78	4	29.27				19.3
PVS	P21266	Glutathione S-transferase Mu 3*	164.12	5	23.11				26.5
PVS	P12277	Creatine kinase B-type*	162.73	3	12.6				42.6
PVS	Q9NZI8	Insulin-like growth factor 2 mRNA-binding protein 1	158.41	6	14.21				63.4
PVS	Q04864	Proto-oncogene c-Rel	157.09	7	11.47				68.5
PVS	P62081	40S ribosomal protein S7	151.23	6	27.32				22.1
PVS	P60866	40S ribosomal protein S20	148.89	4	28.57				13.4
PVS	Q9UI30	tRNA methyltransferase 112 homolog	144.94	2	21.6				14.2
PVS	P40222	Alpha-taxilin*	144.35	1	3.48				61.9
PVS	P84098	60S ribosomal protein L19	142.14	2	12.27				23.5
PVS	Q9C0F1	Centrosomal protein of 44 kDa	140.21	3	12.82				44.1
PVS	P63167	Dynein light chain 1, cytoplasmic	140.02	2	38.2				10.4
PVS	Q9ULW0	Targeting protein for Xklp2	136.48	3	5.09				85.6

PVS	Q8NCA5	Protein FAM98A	134.84	2	5.2				55.4
PVS	Q6FI81	Anamorsin	132.96	3	11.54				33.6
PVS	Q9NZT1	Calmodulin-like protein 5	131.94	2	21.23				15.9
PVS	Q8NHV4	Protein NEDD1	130.42	1	2.73				71.9
PVS	Q9NXV2	BTB/POZ domain-containing protein KCTD5	127.97	2	10.68				26.1
PVS	P62995	Transformer-2 protein homolog beta	126.73	2	12.85				33.6
PVS	P14174	Macrophage migration inhibitory factor*#	126.14	2	17.39				12.5
PVS	Q15717	ELAV-like protein 1	122.58	1	3.37				36.1
PVS	P30740	Leukocyte elastase inhibitor*	119.1	3	6.33				42.7
PVS	P42677	40S ribosomal protein S27*	112.12	2	25				9.5
PVS	P62699	Protein yippee-like 5	110.96	2	21.49				13.8
PVS	Q02878	60S ribosomal protein L6	110.87	4	10.76				32.7
PVS	P32969	60S ribosomal protein L9	108.54	2	5.73				21.8
PVS	P23258	Tubulin gamma-1 chain	104.48	3	8.65				51.1
PVS	Q9H4H8	Protein FAM83D	103.31	2	5.47				64.4
PVS	Q15654	Thyroid receptor-interacting protein 6	103.23	2	5.04				50.3
PVS	Q9BRJ7	Protein syndesmos*	102.77	2	13.27				23.3
PVS	P42766	60S ribosomal protein L35	101.06	1	9.09				14.5
PVS	O00425	Insulin-like growth factor 2 mRNA-binding protein 3	91.68	5	12.09				63.7
PVS	O00743	Serine/threonine-protein phosphatase 6 catalytic subunit*	83.39	2	7.87				35.1
PVS	P46776	60S ribosomal protein L27a	82.71	1	7.43				16.6
PVS	O15479	Melanoma-associated antigen B2	78.75	3	8.46				35.3
PVS	P24666	Low molecular weight phosphotyrosine protein phosphatase	77.56	2	13.29				18
PVS	P22735	Protein-glutamine gamma-glutamyltransferase K (Tgase K)	76.52	2	3.43				89.7
PVS	P62753	40S ribosomal protein S6*	75	1	3.21				28.7
PVS	P15531	Nucleoside diphosphate kinase A	75	4	30.92				17.1
PVS	P60953	Cell division control protein 42 homolog	71.77	1	5.24				21.2
PVS	P69905	Hemoglobin subunit alpha	71.29	1	10.56				15.2
PVS	Q9Y3B4	Pre-mRNA branch site protein p14	68.2	3	24.8				14.6
PVS	P30041	Peroxiredoxin-6#	54.67	2	9.38				25
PVS	P62304	Small nuclear ribonucleoprotein E*	54.38	2	25				10.8
PVS	P59998	Actin-related protein 2/3 complex subunit 4	51.17	1	6.55				19.7
Both	P08670	Vimentin*	1583.4	30	57.51	1101.13	18	41.42	53.6
Both	P15924	Desmoplakin*	1462.36	26	9.68	904.2	16	5.68	331.6
Both	Q16555	Dihydropyrimidinase-related protein 2	1438.95	18	41.78	588.49	8	17.31	62.3
Both	Q02413	Desmoglein-1	1309.94	14	17.25	828.52	10	12.87	113.7
Both	Q99873	Protein arginine N-methyltransferase 1*	1131.09	18	46.26	651.22	10	28.81	41.5
Both	P07437	Tubulin beta chain	923.64	14	31.53	443.5	8	20.27	49.6
Both	Q86YZ3	Hornerin	875.39	10	11.09	344.15	9	10.07	282.2
Both	P14923	Junction plakoglobin#	865.04	18	27.79	695.63	13	17.45	81.7
Both	P68371	Tubulin beta-4B chain	744.63	12	31.46	410.54	8	20.22	49.8

Both	P04075	Fructose-bisphosphate aldolase A	662.92	11	40.11	217.23	6	18.13	39.4
Both	P06733	Alpha-enolase*	648.19	10	27.65	291.53	5	14.98	47.1
Both	P07900	Heat shock protein HSP 90-alpha*#	624.98	18	23.77	729.59	13	16.94	84.6
Both	P08238	Heat shock protein HSP 90-beta#	615.53	13	22.79	646.01	9	24.23	83.2
Both	P62805	Histone H4	578.43	8	43.69	128.02	3	29.13	11.4
Both	Q8WUH6	UPF0444 transmembrane protein C12orf23	492.6	5	57.76	59.23	2	21.55	11.7
Both	P30050	60S ribosomal protein L12	489.22	5	43.03	113.33	2	14.55	17.8
Both	Q6UWP8	Suprabasin	425.4	1	18.31	385.53	2	20.68	60.5
Both	P31944	Caspase-14	410.03	5	17.77	346.38	4	17.36	27.7
Both	P29508	Serpin B3	406.75	5	17.69	334.2	4	12.31	44.5
Both	P18124	60S ribosomal protein L7	391.04	11	37.1	149.17	5	23.39	29.2
Both	P62241	40S ribosomal protein S8	373.54	4	24.52	143.18	2	12.02	24.2
Both	Q01469	Fatty acid-binding protein, epidermal	366.12	4	37.04	144.98	2	18.52	15.2
Both	P60174	Triosephosphate isomerase*#	362	4	18.88	131.77	1	4.2	30.8
Both	P11021	78 kDa glucose-regulated protein	359.83	5	10.55	363.95	6	12.84	72.3
Both	P68366	Tubulin alpha-4A chain	356.55	6	18.08	107.76	4	10.94	49.9
Both	P62888	60S ribosomal protein L30*	349.7	5	56.52	52.38	1	10.43	12.8
Both	Q13642	Four and a half LIM domains protein 1	344.46	5	18.89	198.71	3	11.15	36.2
Both	Q07020	60S ribosomal protein L18	343.41	6	31.38	99.56	2	12.77	21.6
Both	P02788	Lactotransferrin	342.34	8	16.34	826.45	19	33.52	78.1
Both	P26373	60S ribosomal protein L13	328.08	8	28.91	162.94	3	13.27	24.2
Both	P62937	Peptidyl-prolyl cis-trans isomerase A*#	324.92	7	44.24	53.2	1	10.43	18
Both	P08107	Heat shock 70 kDa protein 1A/1B*	321.7	5	10.45	264.91	4	8.42	70
Both	Q92841	Probable ATP-dependent RNA helicase DDX17	313.61	7	12.21	236.46	5	8.09	80.2
Both	P13639	Elongation factor 2*#	311.91	5	6.53	332.65	7	8.28	95.3
Both	P02787	Serotransferrin	286.8	4	7.31	52.19	1	1.58	77
Both	P22234	Multifunctional protein ADE2*	273.61	11	24.47	123.42	9	24	47
Both	P25311	Zinc-alpha-2-glycoprotein	269.54	3	11.41	376.31	3	11.41	34.2
Both	P47929	Galectin-7	255.5	2	18.38	122.43	1	8.09	15.1
Both	Q15365	Poly(rC)-binding protein 1*	253.79	6	22.75	145.3	3	11.8	37.5
Both	O43175	D-3-phosphoglycerate dehydrogenase*#	252.19	6	13.13	756.29	7	14.82	56.6
Both	P46783	40S ribosomal protein S10	247.78	3	14.55	68.16	1	8.48	18.9
Both	P05089	Arginase-1	239.65	5	16.46	101.56	4	13.04	34.7
Both	Q96KK5	Histone H2A type 1-H*	237.37	4	35.95	64.92	1	7.03	13.9
Both	P62913	60S ribosomal protein L11	237.35	3	18.54	267.56	1	7.87	20.2
Both	Q13835	Plakophilin-1	234.76	5	6.96	95.92	3	4.55	82.8
Both	P68871	Hemoglobin subunit beta	233.43	3	28.57	142.39	1	8.84	16
Both	O95544	NAD kinase	227.15	5	10.99	126.68	3	8.52	49.2
Both	P00338	L-lactate dehydrogenase A chain*#	223.75	4	12.65	124.06	4	11.75	36.7
Both	P14618	Pyruvate kinase isozymes M1/M2#	223.74	5	15.07	165.92	3	6.21	57.9
Both	Q14498	RNA-binding protein 39	220.68	4	10	188.79	4	10	59.3

Both	P0CG48	Polyubiquitin-C	220.18	2	32.85	109.2	1	21.02	77
Both	P53999	Activated RNA polymerase II transcriptional coactivator p15	219	5	32.28	99.31	3	20.47	14.4
Both	P21291	Cysteine and glycine-rich protein 1*	217.23	2	16.58	87.04	1	7.77	20.6
Both	P63104	14-3-3 protein zeta/delta	209.74	5	23.27	99.95	3	13.88	27.7
Both	P61978	Heterogeneous nuclear ribonucleoprotein K*#	202.94	3	9.94	615.3	8	22.03	50.9
Both	P50990	T-complex protein 1 subunit theta	201.47	3	6.75	211.81	2	4.56	59.6
Both	Q06830	Peroxiredoxin-1*#	199.37	5	24.12	77.04	3	14.07	22.1
Both	P62917	60S ribosomal protein L8	195.06	1	4.28	121.87	1	4.28	28
Both	P01040	Cystatin-A	191.23	4	66.33	140.85	1	12.24	11
Both	O75223	Gamma-glutamylcyclotransferase	187.65	3	18.09	184.06	4	23.4	21
Both	P15311	Ezrin	185.66	4	6.83	315.78	6	10.75	69.4
Both	Q14247	Src substrate cortactin	185.58	5	10.55	84.38	2	3.82	61.5
Both	P39023	60S ribosomal protein L3	184.56	2	5.21	102.45	3	6.95	46.1
Both	Q02543	60S ribosomal protein L18a	182.14	2	15.34	89.54	1	7.39	20.7
Both	Q15366	Poly(rC)-binding protein 2*	181.26	3	12.33	105.3	3	11.8	38.6
Both	Q07955	Serine/arginine-rich splicing factor 1	179.92	2	8.87	120.53	4	15.73	27.7
Both	P04040	Catalase	177.76	3	9.3	224.73	2	4.17	59.7
Both	Q13247	Serine/arginine-rich splicing factor 6	173.8	5	15.12	114.81	2	5.23	39.6
Both	P00390	Glutathione reductase, mitochondrial	170.65	4	10.92	135.52	2	4.6	56.2
Both	P41091	Eukaryotic translation initiation factor 2 subunit 3#	160.76	5	12.08	94.61	4	11.23	51.1
Both	Q9H6Z4	Ran-binding protein 3	158.09	5	11.64	532	11	25.93	60.2
Both	P61981	14-3-3 protein gamma*	155.22	3	10.93	122.37	2	8.91	28.3
Both	Q2TAM9	Tumor suppressor candidate gene 1 protein	154.03	2	12.74	112.04	2	11.32	23.4
Both	P07339	Cathepsin D*	153.51	3	8.98	264.9	5	15.78	44.5
Both	O95816	BAG family molecular chaperone regulator 2	152.44	5	23.7	166.24	6	28.44	23.8
Both	P17987	T-complex protein 1 subunit alpha*#	149.98	5	9.89	199.19	6	11.87	60.3
Both	P62316	Small nuclear ribonucleoprotein Sm D2*#	148.81	3	24.58	70.16	2	16.95	13.5
Both	P60900	Proteasome subunit alpha type-6	147.83	1	5.28	98.28	1	5.28	27.4
Both	P46781	40S ribosomal protein S9	146.72	4	18.04	110.56	2	10.31	22.6
Both	Q8IVT2	Mitotic interactor and substrate of PLK1	142.36	2	4.42	58.51	2	4.42	75.3
Both	P62277	40S ribosomal protein S13	142.06	5	27.15	79.44	1	6.62	17.2
Both	P40429	60S ribosomal protein L13a	138.95	4	19.21	54.35	3	16.26	23.6
Both	P26641	Elongation factor 1-gamma*	133.39	1	2.97	81.09	1	2.97	50.1
Both	P27348	14-3-3 protein theta*	132.88	3	11.02	54.7	2	8.98	27.7
Both	P29401	Transketolase*	128.84	3	8.03	91.98	2	6.42	67.8
Both	Q08188	Protein-glutamine gamma-glutamyltransferase E (Tgase E)	126.77	4	7.79	104.22	4	8.08	76.6
Both	O75330	Hyaluronan mediated motility receptor	125.32	3	6.91	140.73	3	5.66	84
Both	P12956	X-ray repair cross-complementing protein 6	120	1	2.3	56.14	1	2.3	69.8
Both	Q96IJ6	Mannose-1-phosphate guanyltransferase alpha	119.95	2	5.95	94.21	3	9.29	46.3
Both	P49368	T-complex protein 1 subunit gamma	115.37	3	5.5	220.52	3	5.5	60.5
Both	P48643	T-complex protein 1 subunit epsilon*	107.17	2	3.7	142.22	3	4.99	59.6

Both	Q96P63	Serpin B12	103.85	5	12.84	107.02	3	8.15	46.2
Both	O00154	Cytosolic acyl coenzyme A thioester hydrolase	92.27	2	6.05	155.64	4	12.11	41.8
Both	Q9UJ41	Rab5 GDP/GTP exchange factor	90.48	1	1.41	151.98	4	8.05	79.3
Both	Q765P7	MTSS1-like protein	84.38	2	6.02	220.22	8	15.8	79.9
Both	P28072	Proteasome subunit beta type-6	83.51	2	8.79	68.64	2	8.79	25.3
Both	P06702	Protein S100-A9	80.85	4	32.46	60.29	2	24.56	13.2
Both	P32119	Peroxiredoxin-2	79.08	3	15.15	59.44	1	4.55	21.9
Both	P63244	Guanine nucleotide-binding protein subunit beta-2-like 1	78.4	2	7.89	65.42	1	3.79	35.1
Both	Q9Y2T3	Guanine deaminase	74.92	1	2.64	134.11	1	2.64	51
Both	P12268	Inosine-5'-monophosphate dehydrogenase 2*#	54.8	2	4.47	1045.16	14	29.96	55.8
Both	P60842	Eukaryotic initiation factor 4A-I*	54.23	1	2.46	201.83	2	5.91	46.1
Both	Q99832	T-complex protein 1 subunit eta*	53.82	2	2.58	50.75	3	4.97	59.3
PVSN	Q9Y265	RuvB-like 1				367.21	10	25.88	50.2
PVSN	P20839	Inosine-5'-monophosphate dehydrogenase 1				347.48	6	11.87	55.4
PVSN	Q9Y230	RuvB-like 2#				338.45	10	22.68	51.1
PVSN	P13716	Delta-aminolevulinic acid dehydratase				292.8	6	22.73	36.3
PVSN	Q15276	Rab GTPase-binding effector protein 1				271.41	7	9.86	99.2
PVSN	P49862	Kallikrein-7				189.7	3	12.65	27.5
PVSN	O60825	6-phosphofructo-2-kinase/fructose-2,6-bisphosphatase 2*				172.16	4	8.12	58.4
PVSN	Q9H479	Fructosamine-3-kinase				142.54	2	8.41	35.1
PVSN	O14744	Protein arginine N-methyltransferase 5*				129.83	4	6.44	72.6
PVSN	O00299	Chloride intracellular channel protein 1*				106.2	3	13.69	26.9

*: Low pKa

#: Oxidation

Supplementary Table S2. Identified Modification Sites of PVS-tagged Proteins

Protein Species	Accession #	Sequence	Score
Vimentin*	P08670	QVQSLT**C**EVDALK	69
Protein arginine N-methyltransferase 1*	Q99873	VIGIE**C**SSISDYAVK	68
Dihydropyrimidinase-related protein 2	Q16555	GLYDGPV**C**EVSVTPK	66
Pyrroline-5-carboxylate reductase 3	Q53H96	AATMSAVEAAT**C**R	62
40S ribosomal protein S3*	P23396	GL**C**AIAQAESLR	56
WD repeat-containing protein 91	A4D1P6	ELFSTTTSQ**C**AEK	55
Anamorsin	Q6FI81	SA**C**GN**C**YLGDAFR	52
Pyrroline-5-carboxylate reductase 3	Q53H96	AATMSAVEAAT**C**R	48
Hyaluronan mediated motility receptor	O75330	FNDPSG**C**APSPGAYDVK	48
Transcriptional activator protein Pur-beta	Q96QR8	GGGGGP**C**GFQPASR	48
Transcriptional activator protein Pur-beta	Q96QR8	FFFDVG**C**NK	48
Poly(rC)-binding protein 1*<br>Poly(rC)-binding protein 2	Q15365<br>Q15366	INISEGN**C**PER	45
Sequestosome-1	Q13501	DHRPP**C**AQEAPR	43
Signal recognition particle 9 kDa protein*#	P49458	VTDDLV**C**LVYK	43
Glucose-6-phosphate 1-dehydrogenase*	P11413	TQV**C**GILR	43
Poly(rC)-binding protein 1*<br>Poly(rC)-binding protein 2	Q15365<br>Q15366	LVVPATQ**C**GSLIGK	42
Targeting protein for Xklp2	Q9ULW0	TVEI**C**PFSFDSR	41

Four and a half LIM domains protein 1	Q13642	F**C**ANTCVECR	40
WD repeat-containing protein 91	A4D1P6	KPEASGPEAEP**C**PELHTEPVEPLTR	40
Gametogenetin-binding protein 2	Q9H3C7	GY**C**AALYEGLR	40
Elongation factor 2#	P13639	STLTDSLV**C**K	38
T-complex protein 1 subunit alpha	P17987	I**C**DDELILIK	38
Protein arginine N-methyltransferase 1	Q99873	QLVTNA**C**LIK	38
Tubulin beta chain<br>Tubulin beta-4B chain	P68371<br>P07437	EIVHLQAGQ**C**GNQIGAK	37
Ethanolamine-phosphate phospho-lyase	Q8TBG4	TESVTSENTP**C**K	36
Transcriptional activator protein Pur-beta	Q96QR8	FGGAF**C**R	36
Serine/arginine-rich splicing factor 3	P84103	DS**C**PLDCK	36
RNA-binding protein 47	A0AV96	LLGV**CC**SVDNCR	36
Serine/arginine-rich splicing factor 1#	Q07955	EAGDV**C**YADVYR	35
tRNAmethyltransferase 112 homolog	Q9UI30	I**C**PVEFNPNFVAR	35
Glutathione S-transferase Mu 3*	P21266	IAAYLQSDQF**C**K	35
Round spermatid basic protein 1	Q5VWQ0	ELEVDSQ**C**VR	34
Gephyrin	Q9NQX3	LSTAS**C**PTPK	34
Tubulin beta chain<br>Tubulin beta-4B chain	P07437<br>P68371	TAV**C**DIPPR	34
BTB/POZ domain-containing protein KCTD5	Q9NXV2	GGIGAGLGGGL**C**R	33
Tubulin alpha-1B chain<br>Tubulin alpha-4A chain	P68363<br>P68366	SIQFVDW**C**PTGFK	32

Destrin	P60981	A**C**IAEK	32
Protein SEC13 homolog*	P55735	FASGG**C**DNLIK	31
6-phosphofructokinase type C	Q01813	AA**C**NLLQR	30
Non-POU domain-containing octamer-binding protein*	Q15233	FA**C**HSASLTVR	30
Ankyrin repeat and SAM domain-containing protein 3	Q6ZW76	GQV**C**QEQELR	29
Eukaryotic translation initiation factor 2 subunit 3	P41091	IVLTNPV**C**TEVGEK	29
Transforming acidic coiled-coil-containing protein 2	O95359	**C**AQEYLSR	28
Forkhead box protein K1	P85037	SGGLQTPE**C**LSR	28
60S ribosomal protein L14	P50914	ALVDGP**C**TQVR	28
Peptidyl-prolylcis-trans isomerase A	P62937	KITIAD**C**GQLE	27
Tubulin beta chain*<br>Tubulin beta-4B chain	P07437<br>P68371	NMMAA**C**DPR	26
Heterogeneous nuclear ribonucleoprotein M	P52272	G**C**GVVK	24

*: Reactive cysteine, also found in reference 28.

#: Cysteine prone to oxidation, also found in reference 29.

Supplementary Table S3. Identified Modification Sites of PVSN-tagged Proteins

Protein Species	Accession #	Sequence	Score
Glutathione reductase, mitochondrial*	P00390	LGGT**C**VNVGCVPK	63
Protein arginine N-methyltransferase 1	Q99873	GQLCELS**C**STDYR	61
Fructosamine-3-kinase	Q9H479	AFGGPGAG**C**ISEGR	54
Serine/arginine-rich splicing factor 1#	Q07955	EAGDV**C**YADVYR	53
Dihydropyrimidinase-related protein 2	Q16555	GLYDGPV**C**EVSVTPK	52
Glyceraldehyde-3-phosphate dehydrogenase#	P04406	IISNAS**C**TTNCLAPLAK	49
Four and a half LIM domains protein 1	Q13642	F**C**ANTCVECR	46
Eukaryotic peptide chain release factor GTP-binding subunit ERF3A	P15170	LESGSI**C**K	44
Fructose-bisphosphatealdolase A	P04075	ALANSLA**C**QGK	41
40S ribosomal protein S3*	P23396	GL**C**AIAQAESLR	40
Poly(rC)-binding protein 1*<br>Poly(rC)-binding protein 2	Q15365<br>Q15366	INISEGN**C**PER	40
60S ribosomal protein L18	Q07020	G**C**GTVLLSGPR	38
Inosine-5'-monophosphate dehydrogenase 2*#	P12268	HGF**C**GIPITDTGR	36
Elongation factor 2*#	P13639	STLTDSLV**C**K	35
WD repeat-containing protein 41	Q9HAD4	IS**C**FQSTVK	35
Vimentin*	P08670	QVQSLT**C**EVDALK	34
40S ribosomal protein S2*	P15880	G**C**TATLGNFAK	34
Hyaluronan mediated motility receptor*	O75330	FNDPSG**C**APSPGAYDVK	33

Eukaryotic translation initiation factor 2 subunit 3	P41091	S**C**GSSTPDEFPTDIPGTK	30
40S ribosomal protein S27	P42677	LTEG**C**SFR	30
WD repeat-containing protein 26	Q9H7D7	**C**ELTPLK	30
RuvB-like 2#	Q9Y230	FVQ**C**PDGELQK	30
Armadillo repeat-containing protein 8	Q8IUR7	TDDN**C**IVLK	29
Protein arginine N-methyltransferase 1	Q99873	QLVTNA**C**LIK	29
Calpain small subunit 1#	P04632	TDGFGIDT**C**R	28
Destrin	P60981	A**C**IAEK	28
Chloride intracellular channel protein 1*	O00299	IGN**C**PFSQR	26
T-complex protein 1 subunit alpha	P17987	D**C**LINAAK	25
Aldehyde dehydrogenase family 16 member A1	Q8IZ83	GAAA**C**DLVQR	25
Elongation factor 2#	P13639	**C**ITIK	24
Serine/arginine-rich splicing factor 3	P84103	DS**C**PLDCK	24
RuvB-like 1	Q9Y265	GN**C**VIR	23
Serine/arginine-rich splicing factor 1	Q07955	GPAGNND**C**R	20

*: Reactive cysteine, also found in reference 28.

#: Cysteine prone to oxidation, also found in reference 29.
